# Supplementary material for: Social support and psychosocial well-being among older adults in Europe during the COVID-19 pandemic: a cross-sectional study
Source: BMJ Open. 2023 Jul 5;13(7):e071533. doi: 10.1136/bmjopen-2022-071533 (PMC10335576; doi:10.1136/bmjopen-2022-071533)
Supplement: Supplementary data [file bmjopen-2022-071533supp001.pdf]

Appendix

Table 1a: Sample description (Share 8).

age	Freq.	Percent	Cum.
65-69 years	9857	26.92	26.92
70-74 years	9719	26.54	53.46
75-79 years	7508	20.50	73.96
80-84 years	5399	14.74	88.70
85+	4138	11.30	100.00
Sex	Freq.	Percent	Cum.
Male	15719	42.92	42.92
Female	20902	57.08	100.00
Employment status	Freq.	Percent	Cum.
Retired	32195	88.03	88.03
Employment	1626	4.45	92.48
Unemployed	79	0.22	92.69
Permanently sick or disabled	298	0.81	93.51
Homemaker	1980	5.41	98.92
Other	395	1.08	100.00
Rating of subjective health	Freq.	Percent	Cum.
Excellent	1266	3.46	3.46
Very good	4789	13.09	16.55
Good	14290	39.05	55.60
Fair	12106	33.08	88.69
Poor	4140	11.31	100.00
Country			
Austria	1943	5.31	5.31
Germany	1466	4.00	9.31
Sweden	856	2.34	11.65
Netherlands	592	1.62	13.26
Spain	1545	4.22	17.48
Italy	2528	6.90	24.38
France	1458	3.98	28.37

Denmark	1143	3.12	31.49
Greece	2515	6.87	38.36
Switzerland	1452	3.96	42.32
Belgium	2427	6.63	48.95
Israel	1132	3.09	52.04
Czech Republic	1808	4.94	56.98
Poland	1795	4.90	61.88
Luxembourg	581	1.59	63.46
Hungary	715	1.95	65.42
Portugal	860	2.35	67.76
Slovenia	2313	6.32	74.08
Estonia	3012	8.22	82.31
Croatia	1305	3.56	85.87
Lithuania	774	2.11	87.98
Bulgaria	480	1.31	89.29
Cyprus	490	1.34	90.63
Finland	855	2.33	92.97
Latvia	623	1.70	94.67
Malta	548	1.50	96.16
Romania	919	2.51	98.67
Slovakia	486	1.33	100.00
