# Supplementary material for: Social support and psychosocial well-being among older adults in Europe during the COVID-19 pandemic: a cross-sectional study
Source: BMJ Open. 2023 Jul 5;13(7):e071533. doi: 10.1136/bmjopen-2022-071533 (PMC10335576; doi:10.1136/bmjopen-2022-071533)
Supplement: Author's manuscript [file bmjopen-2022-071533.draft_revisions.pdf]

BMJ Open

BMJ Open is committed to open peer review. As part of this commitment we make the peer review history of every article we publish publicly available.

When an article is published we post the peer reviewers' comments and the authors' responses online. We also post the versions of the paper that were used during peer review. These are the versions that the peer review comments apply to.

The versions of the paper that follow are the versions that were submitted during the peer review process. They are not the versions of record or the final published versions. They should not be cited or distributed as the published version of this manuscript.

BMJ Open is an open access journal and the full, final, typeset and author-corrected version of record of the manuscript is available on our site with no access controls, subscription charges or pay-per-view fees (http://bmjopen.bmj.com).

If you have any questions on BMJ Open's open peer review process please email info.bmjopen@bmj.com

BMJ Open

# Social support and psychosocial well-being among elderly men and women in Europe during the COVID-19 pandemic

| | |
|---|---|
| Journal: | *BMJ Open* |
| Manuscript ID | bmjopen-2022-071533 |
| Article Type: | Original research |
| Date Submitted by the Author: | 13-Jan-2023 |
| Complete List of Authors: | Lu, Ji; Tongji Medical College,<br>Xiong, Juyang; Huazhong University of Science and Technology Tongji Medical College, School of Medicine and Health Management<br>Tang, Shangfeng; School of medicine and health management, tongji mediical college, Huazhong university of sciience and techonolgy, Health mangament<br>Bishwajit, Ghose; University of Ottawa, Faculty of Health Sciences;<br>Guo, Shuyan; National Institute of Hospital Administration, National Health Commission |
| Keywords: | COVID-19, MENTAL HEALTH, SOCIAL MEDICINE |

SCHOLARONE™
Manuscripts

*I, the Submitting Author has the right to grant and does grant on behalf of all authors of the Work (as defined in the below author licence), an exclusive licence and/or a non-exclusive licence for contributions from authors who are: i) UK Crown employees; ii) where BMJ has agreed a CC-BY licence shall apply, and/or iii) in accordance with the terms applicable for US Federal Government officers or employees acting as part of their official duties; on a worldwide, perpetual, irrevocable, royalty-free basis to BMJ Publishing Group Ltd ("BMJ") its licensees and where the relevant Journal is co-owned by BMJ to the co-owners of the Journal, to publish the Work in this journal and any other BMJ products and to exploit all rights, as set out in our licence.*

*The Submitting Author accepts and understands that any supply made under these terms is made by BMJ to the Submitting Author unless you are acting as an employee on behalf of your employer or a postgraduate student of an affiliated institution which is paying any applicable article publishing charge ("APC") for Open Access articles. Where the Submitting Author wishes to make the Work available on an Open Access basis (and intends to pay the relevant APC), the terms of reuse of such Open Access shall be governed by a Creative Commons licence – details of these licences and which Creative Commons licence will apply to this Work are set out in our licence referred to above.*

*Other than as permitted in any relevant BMJ Author's Self Archiving Policies, I confirm this Work has not been accepted for publication elsewhere, is not being considered for publication elsewhere and does not duplicate material already published. I confirm all authors consent to publication of this Work and authorise the granting of this licence.*

For peer review only

# Social support and psychosocial well-being among elderly men and women in Europe during the COVID-19 pandemic

**Lu Ji[1*], PhD; Juyang Xiong[1*], PhD; Shangfeng Tang[1], PhD; Ghose Bishwajit[2], PhD; Shuyan Guo[3]**

1 Lu Ji， School of Medicine and Health Management, Tongji Medical College of Huazhong University of Science and Technology, Wuhan, China. Jilu2020@hust.edu.cn

1 Juyang Xiong, School of Medicine and Health Management, Tongji Medical College of Huazhong University of Science and Technology, Wuhan, China. xiongjuyang@hust.edu.cn

1 Shangfeng Tang, School of Medicine and Health Management, Tongji Medical College of Huazhong University of Science and Technology, Wuhan, China. Sftang2018@hust.edu.cn

2 Ghose Bishwajit, Faculty of Health Sciences, University of Ottawa, Ottawa, Ontario, Canada. Brammaputram@gmail.com

3 Shuyan Guo, National Institute of Hospital Administration, National Health Commission, Beijing, China, guoshuyan@niha.org.cn

*Lu Ji and Juyang Xiong contributed equally

For peer review only

Correspondence to Shuyan Guo, guoshuyan@niha.org.cn

Word count: 3800.

For peer review only

**Abstract**: The objective of the study was to identify the association between social support and psychosocial well-being among men and women aged over 65 years in Europe during the COVID-19 pandemic.

**Methods**: Cross-sectional data on 36,621 men (n=15,719) and women (n=20,902) aged 65 years or higher were obtained from the ninth round of the Survey of Health, Ageing and Retirement in Europe (SHARE). The outcomes were measured by psychosocial well-being reflected with self-reported depression, nervousness, loneliness and sleep disturbances. Social support was measured in terms of receiving help from own children, relatives, and neighbours/friends/colleagues since the pandemic outbreak.

**Result**: About one-third of the participants reported depression (31.03%), nervousness (32.85%), loneliness (32.23%), and sleep trouble (33.01%). The results of multivariable regression analysis revealed that social support was a protective factor to psychological well-being. For instance, receiving help from own children [RD= -0.13, 95% CI = -0.14, -0.12], relatives [RD= -0.08, 95% CI = -0.11, -0.06], neighbours/friends/colleagues [RD= -0.11, 95% CI = -0.13, -0.09], and receiving home care [RD= -0.20, 95% CI = -0.22, -0.18] showed significantly lower risk difference for depression. Similar findings were noted for loneliness, nervousness, and sleep trouble as well, with the risk difference being slightly different for men and women in the gender-stratified analysis. For instance, the risk difference in depression for receiving help from own children was -0.10 [95% confidence interval = -0.12, -0.08] among men compared with -0.12 [95% confidence interval = -0.14, -0.11] among women. The risk differences in the outcome measures were calculated using Generalized Linear Model for binomial family.

**Conclusion**: Findings of the present study highlight a protective role of social support on psychological well-being among both men and women. Developing strategies to promote social support, especially among elderly individuals, may mitigate the rising burden of psychological illness during the COVID-19 pandemic.

**Keywords**: COVID-19; Elderly health; Mental health; Social support; Well-being.

For peer review only

## Introduction

The COVID-19 pandemic has become one of the most devastating health and social crises in generations due to its long-term impacts on physical and mental health, social life and livelihoods worldwide.[1–3] A pervasive crisis is the emergence of a phenomenon known as "coronaphobia" - fear or anxiety related to the virus and its potential impacts on the psychosocial well-being of individuals and communities at large.[4, 5] A growing number of studies have highlighted the prevalence of mental illness in the context of lifestyle such as higher consumption of psychoactive drugs and widespread social isolation and loneliness.[6–9] The fear of contracting the virus has forced people stay at home for a long period of time, which leads more severe among elderly individuals who share a heightened risk of severe illness from the virus.[10]

The measures to controll COVID-19 pandemic by physical distancing have caused disruptions a healthy and active lifestyle, and the opportunities to maintain social connections and seek help in times of need.[11] The impact of social isolation and loneliness on mental health has been an increasing concern in the wake of the global pandemic, as socialization opportunities have become limited and physical distancing rules have been put in place.[12] As such, there has been a dramatic decrease in social interaction and support, which is particularly concerning for peoples' psychosocial well-being during the global health crisis. Prolonged isolation can lead to feelings of loneliness, depression and anxiety and can take a toll on mental health of elderly individuals due to their higher dependence and physical and emotional care.

The feeling of depression and loneliness are common mental health concerns that can affect people from all walks of life. However, studies have revealed that elderly people are more likely to suffer from mental health issues than their younger counterparts.[13] This is due to a range of factors, including changes in physical health and lifestyle, increased isolation and loneliness, financial concerns, and difficulty in adapting to the technological advances of modern life. Furthermore, elderly individuals are less opportunities to talk about their feelings or seek help for

For peer review only

depression due to stigma or embarrassment[14], and are also less likely to be able to socialize and participate in activities owing to more frequent illness episodes and decreased mobility.[15]

The COVID-19 pandemic has fuelled social isolation and loneliness mainly through fewer opportunities for meeting friends and families, organizing or participating in social gatherings and reduced social support.[16] Social support, which is a key aspect of social capital, has been shown to be strongly correlated with mental health in previous studies.[17, 18] Perceived social support refers to the subjective availability of psychological, physical, and financial help from social relationships such as family, friends, neighbours, and community members that is available in times of need. Social support plays an important role in mitigating the impact of social isolation and can help reduce stress, improve mood, and promote positive coping mechanisms.[19] Social support also provides a sense of belonging and connection, which can offset feelings of loneliness and isolation. Several previous studies have demonstrated the link between social relationships and support and mental health.[17, 19, 20] However, there is not enough evidence regarding this relationship among elderly individuals in European countries in the context of the COVID-19 pandemic. The present study therefore investigates the relationship between social support and psychosocial well-being among elderly men and women in Europe using the data from the Survey of Health Ageing, and Retirement (WAVE 9).

For peer review only

## Methods

### Data source

Data for this study were derived from the ninth wave of the Survey of Health Ageing, and Retirement in Europe (SHARE).[21] The survey aims to provide a framework for researchers to better understand population ageing and covers most of the European Union and Israel. SHARE has been adopted as a model by a number of ageing surveys throughout the world and is harmonized with the US Health and Retirement Study (HRS) and the English Longitudinal Study of Ageing (ELSA).[22] The SHARE survey collects data on a diverse range of health and socioeconomic indicators among individuals including demographics, living conditions, income and employment status, mental health, satisfaction with health conditions and social networks. The ninth wave of the Survey included questions relevant to COVID-19, such as receiving support from social connections to obtain basic necessities. Data for this survey were collected via computer-assisted telephone interviews between June and August of 2021 among individuals aged 50 or older. For the purpose of the present study, the analysis was limited to participants aged 65 years or older only.

For peer review only

### Description of the variables

The outcome measures included four indicators of psychosocial well-being assessed iby self-reported nervousness, depression, sleep disturbances and loneliness. These indicators were assessed by the following questions: 1) felt nervous in last month (Yes/No), 2) sad or depressed in last month (Yes/No), 3) trouble sleeping recently (Yes/No), 4) how often feelings of loneliness (Often/Some of the times/Hardly ever or never). For loneliness, the answers were recoded to 'Yes' for responses 'Often/Some of the times' and to 'No' for responses 'Hardly ever or never'. Social support was assessed in terms of receiving help in obtaining necessities during the pandemic from own children, relatives, and neighbours/friends/colleagues to obtain necessities since the outbreak. These indicators were assessed by the following questions: 1) help received from own children to obtain necessities since the outbreak (Yes/No), 2) help received from other relatives to obtain necessities since the outbreak (Yes/No), 3) help received from neighbours/friends/colleagues to obtain necessities (Yes/No), 4) received regular home care during last 3 months (Yes/No). Apart from the indicators of social support, the analysis also included several socioeconomic variables based on their known association with mental health status such as age (65-69 years/ 70-74 years/ 75-79 years/ 80-84 years/ 85+ years), sex (male/female), employment situation (Retired/ Employed or self-employed/ Unemployed/ Permanently sick or disabled? Homemaker/ Other), and country of residence (list of countries provided in appendix).

**Statistical Analysis**

For peer review only

All statistical analyses were conducted using Stata 17 (Stata Corp., College Station, Texas). The dataset was first examined to make sure the sample population was defined correctly (65 years and older) and there were no missing values and outliers. Descriptive statistics were carried out to calculate the percentage of participants reporting nervousness, depression, loneliness, and sleep trouble. Sex and country-level percentages were presented as bar charts. In the next step, we conducted generalized linear models (GLMs) for binomial family to measure the risk difference (with 95% confidence intervals) in the four outcome variables (nervousness, depression, loneliness, and sleep trouble). The results of GLMs were presented as risk difference (RD), defined as the risk of a condition between an exposed group and an unexposed group.[23] A positive RD value indicates a higher risk by the exposure, whereas a negative one indicates a lower risk. Given the well-documented gender differences in mental health outcomes, the analysis was stratified by male and female participants for each outcome variable. The level of statistical significance for all associations was set at 0.05.

## Results

The sociodemographic characteristics of the sample population are available in the appendix file. A greater percentage of the participants were in the age bracket of 65-69 years (n= 26.92%), female (n= 57.08%), retired (n= 88.03%), and rated general health status as good (n= 39.05%).

Sample size for the participating countries was as follows: Austria (n= 1943), Germany (n= 1466), Sweden (n= 856), Netherlands (n= 592), Spain (n= 1545), Italy (n= 2528), France (n= 1458),

For peer review only

Denmark (n= 1143), Greece (n= 2515), Switzerland (n= 1452), Belgium (n= 2427), Israel (n= 1132), Czech Republic (n= 1808), Poland (n= 1795), Luxembourg (n= 581), Hungary (n= 715), Portugal (n= 860), Slovenia (n= 2313), Estonia (n= 3012), Croatia (n= 1305), Lithuania (n= 774), Bulgaria (n= 480), Cyprus (n= 490), Finland (n= 855), Latvia (n= 623), Malta (n= 548), Romania (n= 919), Slovakia (n= 486).

Among the four psychosocial well-being indicators, sleep trouble (33.01%) was the most commonly reported one (table 1), followed by nervousness (32.85%) and loneliness (32.23%). As described in table 1, the percentage of reporting depression (77.08% vs 62.88%), nervousness (73.56% vs 62.33%), and loneliness (76.43% vs 61.27%) was higher in men, while that of sleep trouble was higher in women (25.73% vs 38.49%).

**Table 1: Percentage of participants who reported having depression, Nervousness, Loneliness, and Sleep Trouble by sex.**

| | Percentage of Total | Percentage of total Men | Percentage of total Women |
|---|---|---|---|
| Depression | 31.03 | 22.92 | 37.12 |
| Nervousness | 32.85 | 26.44 | 37.67 |

For peer review only

| | | | |
|---|---|---|---|
| Loneliness | 32.23 | 23.57 | 38.73 |
| Sleep trouble | 33.01 | 25.73 | 38.49 |

**Figure 1: Percentage of participants who reported having depression, Nervousness, Loneliness, and Sleep Trouble by country.**

For peer review only

**Figure 2: comparison of the total number of conditions reported by male and female participants.**

For peer review only

The results of multivariable regression analysis on the association between self-reported depression and social support are shown in table 2. Receiving help from own children [RD= -0.13, 95% confidence interval = -0.14, -0.12], relatives [RD= -0.08, 95% confidence interval = -0.11, -0.06], neighbours/friends/colleagues [RD= -0.11, 95% confidence interval = -0.13, -0.09], and receiving home care [RD= -0.20, 95% confidence interval = -0.22, -0.18] showed significantly lower risk difference for depression, with the differences being slightly higher among women compared with men. For instance, the risk difference in depression for receiving help from own children was -0.10 [95% confidence interval = -0.12, -0.08] among men compared with -0.12 [95% confidence interval = -0.14, -0.11] among women.

Table 2: Association between social support and self-reported depression.

| | **Full sample** | **Men** | **Women** |
|---|---|---|---|
| **Received help from own children (Yes)** | ref | ref | ref |
| No | -0.13*** [-0.14,-0.12] | -0.10*** [-0.12,-0.08] | -0.12*** [-0.14,-0.11] |
| **Received help from relatives (Yes)** | ref | ref | ref |
| No | -0.08*** [-0.11,-0.06] | -0.07*** [-0.11,-0.03] | -0.08*** [-0.11,-0.05] |
| **Received help from neighbors/friends/colleagues (yes)** | ref | ref | ref |
| No | -0.11*** [-0.13,-0.09] | -0.09*** [-0.12,-0.06] | -0.11*** [-0.13,-0.08] |
| **Received home care (Yes)** | ref | ref | ref |
| No | -0.20*** [-0.22,-0.18] | -0.19*** [-0.22,-0.15] | -0.19*** [-0.21,-0.16] |

For peer review only

N.B. cells represent risk differences with 95% confidence intervals. All models are adjusted for age, sex, employment status, subjective health status, and country of residence. Level of significance: $^{*}$ $p < 0.05$, $^{**}$ $p < 0.01$, $^{***}$ $p < 1$

The results of multivariable regression analysis on the association between social support and self-reported nervousness are shown in table 3. Receiving help from own children [RD= -0.10, 95% confidence interval = -0.12, -0.09], relatives [RD= -0.05, 95% confidence interval = -0.07, -0.03], neighbours/friends/colleagues [RD= -0.08, 95% confidence interval = -0.10, -0.06], and receiving home care [RD= -0.15, 95% confidence interval = -0.17, -0.13] showed significantly lower risk difference for nervousness. In the gender-stratified analysis, receiving help from own children showed a relatively higher risk difference among women, whereas receiving help from neighbours/friends/colleagues showed a relatively higher risk difference among men.

**Table 3:** Association between social support and self-reported nervousness.

| | **Full sample** | Men | Women |
|---|---|---|---|
| **Received help from own children (Yes)** | ref | ref | ref |

| | | | |
|---|---|---|---|
| No | $-0.10^{***}$ [-0.12,-0.09] | $-0.10^{***}$ [-0.11,-0.08] | $-0.09^{***}$ [-0.11,-0.08] |
| **Received help from relatives (Yes)** | ref | ref | ref |
| No | $-0.05^{***}$ [-0.07,-0.03] | $-0.04^{*}$ [-0.08,-0.01] | $-0.05^{***}$ [-0.08,-0.02] |
| **Received help from neighbors/friends/colleagues (yes)** | ref | ref | ref |
| No | $-0.08^{***}$ [-0.10,-0.06] | $-0.06^{***}$ [-0.09,-0.02] | $-0.09^{***}$ [-0.11,-0.06] |
| **Received home care (Yes)** | ref | ref | ref |
| No | $-0.15^{***}$ [-0.17,-0.13] | $-0.15^{***}$ [-0.18,-0.11] | $-0.15^{***}$ [-0.17,-0.12] |

N.B. cells represent risk differences with 95% confidence intervals. All models are adjusted for age, sex, employment status, subjective health status, and country of residence. Level of significance: $^{*}$ $p < 0.05$, $^{**}$ $p < 0.01$, $^{***}$ $p < 1$

The results of multivariable regression analysis on the association between social support and loneliness are shown in table 4. Receiving help from own children [RD= -0.13, 95% confidence interval = -0.14, -0.11] relatives [RD= -0.08, 95% confidence interval = -0.10,-0.05]

For peer review only

neighbours/friends/colleagues[RD= -0.12, 95% confidence interval = -0.13, -0.10], and receiving home care [RD= -0.12, 95% confidence interval = -0.14,-0.10] showed significantly lower risk difference for loneliness. The risk difference was relatively higher among men who reported receiving help from own children, relatives, and neighbours/friends/colleagues.

For peer review only

**Table 4:** Association between social support and self-reported loneliness.

| | Full sample | Men | Women |
|---|---|---|---|
| **Received help from own children (Yes)** | ref | ref | ref |
| No | -0.13*** [-0.14,-0.11] | -0.09*** [-0.11,-0.08] | -0.12*** [-0.13,-0.10] |
| **Received help from relatives (Yes)** | ref | ref | ref |
| No | -0.08*** [-0.10,-0.05] | -0.07*** [-0.11,-0.03] | -0.08*** [-0.10,-0.05] |
| | | | |

| | | | |
|---|---|---|---|
| **Received help from neighbors/friends/colleagues (yes)** | ref | ref | ref |
| No | -0.12*** [-0.13,-0.10] | -0.07*** [-0.10,-0.04] | -0.12*** [-0.15,-0.10] |
| **Received home care (Yes)** | ref | ref | ref |
| No | -0.12*** [-0.14,-0.10] | -0.13*** [-0.16,-0.10] | -0.10*** [-0.12,-0.07] |

N.B. cells represent risk differences with 95% confidence intervals. All models are adjusted for age, sex, employment status, subjective health status, and country of residence. Level of significance: * $p < 0.05$, ** $p < 0.01$, *** $p < 1$

For peer review only

The results of multivariable regression analysis on the association between social support and sleep trouble are shown in table 5. Receiving help from own children [RD= -0.10, 95% confidence interval = -0.11,-0.09], relatives [RD= -0.05, 95% confidence interval = -0.08, -0.03], neighbours/friends/colleagues [-0.07, 95% confidence interval = -0.09,-0.05], and receiving home care [RD= -0.12, 95% confidence interval =-0.14,-0.10] showed significantly lower risk difference for sleep trouble. In the gender-stratified analysis, receiving help from own children and from neighbours/friends/colleagues showed a relatively higher risk difference among men.

For peer review only

**Table 5:** Association between social support and sleep trouble.

| | Full sample | Men | Women |
|---|---|---|---|
| **Received help from own children (Yes)** | ref | ref | ref |
| No | -0.10*** [-0.11,-0.09] | -0.07*** [-0.09,-0.05] | -0.09*** [-0.10,-0.08] |
| | | | |

| | | | |
|---|---|---|---|
| **Received help from relatives (Yes)** | ref | ref | ref |
| No | -0.05*** [-0.08,-0.03] | -0.06** [-0.10,-0.02] | -0.04** [-0.07,-0.01] |
| **Received help from neighbors/friends/colleagues (yes)** | ref | ref | ref |
| No | -0.07*** [-0.09,-0.05] | -0.06*** [-0.09,-0.03] | -0.07*** [-0.09,-0.04] |
| **Received home care (Yes)** | ref | ref | ref |
| No | -0.12*** [-0.14,-0.10] | -0.13*** [-0.16,-0.10] | -0.10*** [-0.12,-0.07] |

N.B. cells represent risk differences with 95% confidence intervals. All models are adjusted for age, sex, employment status, subjective health status, and country of residence. Level of significance: * $p < 0.05$, ** $p < 0.01$, *** $p < 1$

**Discussion**

For peer review only

Given the rising burden of mental illness during the COVID-19 pandemic, it's important to explore the potential role of the social determinants that can affect mental health, especially among elderly individuals who share a greater vulnerability to both COVID-19 and mental health conditions. The present study aimed to assess the relationship between social support and indicators of psychosocial well-being among elderly men and women in 27 countries in Europe and Israel using data from the Survey of Health Ageing, and Retirement. Our findings suggest that about one-third of the participants reported depression, nervousness, loneliness, and sleep trouble, with the percentage of all four conditions being relatively higher among women. Overall, about a quarter of the participants reported at least one, and about 8% reported having all of the four conditions. However, the percentage of reporting multiple conditions was higher among women than among men, indicating a greater burden of psychological issues among women. Previous studies have reported a higher burden of mental health issues among women and attempted to explain the gender differences in mental disorders through various biological (pregnancy, hormonal) and psychosocial factors (self-esteem, social empowerment).[24–26] Apart from the gender differences, we observed remarkable inter-country differences in the corresponding percentages as well. For instance, the percentage of depression ranged from 46.44% in Portugal to 16.7% in Denmark, nervousness from 53.37% in Portugal to 14.53% in the Netherlands, loneliness from 52.91% in Greece to 11.49% in Denmark, and sleep trouble from 49.13% in Estonia to 15.2% in the Netherlands. The country-level disparities might be rooted in the health policies related to the pandemic and health and social conditions that are relevant to mental health in individual countries that should be investigated through more in-depth studies.

For peer review only

For peer review only

Regarding the association between social support in obtaining the necessities during the pandemic and the outcome measures, receiving help from own children, relatives, neighbours/friends/colleagues, and home care showed significantly lower risks of depression, nervousness, loneliness, and sleep trouble compared with those didn't receive any help. Several previous studies have reported the positive association between social support and psychosocial well-being through a social capital lens. According to a cohort study conducted in Quebec, Canada, adolescents who felt they had more social support one year later reported having fewer mental health issues, concluding that perceived social support, especially in those who have mental health issues in youth, may shield against mental health issues as people enter adulthood.[20] A meta-analysis involving 64 studies reported a mean effect size of 0.356, indicating a moderate effect size of social support on mental health.[17] Another study conducted in Lebanon using structured questionnaires for depression (Patient Health Questionnaire), loneliness (UCLA Loneliness Scale) and social support (Multidimensional Scale of Perceived Social Support) found that the rates of depression and loneliness were significantly higher among participants who experienced self-isolation compared to those who didn't.[27] The same study reported that the risk of depressive symptoms and poor sleep quality were respectively 63% and 52% lower among individuals who reported higher levels of social support compared to those with low perceived social support.[27]

The findings of the current study are consistent with previous ones regarding the positive role of social support on psychological well-being. Social support can take many forms, from family and friends to community groups and online forums, and serves the purpose of providing a

For peer review only

sense of connection and belonging and improving coping skills, which can be vital for people who are feeling isolated. Lack of social support can therefore exacerbate the effects of social isolation and lead to further mental health problems. Arguably, the social stressors associated with the pandemic can lead to short- and long-term psychological issues such as anxiety and depression, which in turn can contribute to feelings of loneliness and isolation. With the increasing demands of modern life, individuals are becoming more aware of the protective effects of relationships on health and overall quality of life as it can act as a buffer against social stressors and promote positive mental well-being. Mental health experts have also alarmed about the dangers of social isolation since the start of the COVID-19 pandemic. Policymakers and social researchers should therefore aim to develop innovative strategies for promoting this invaluable resource to build more resilient communities that can help mitigate the long-term mental health impacts of the pandemic.

As far as we are concerned, this is the first study to focus on the association between social support and psychosocial well-being among the elderly population during the COVID-19 pandemic in EU countries. The sample size was large and the data were obtained from a reputed population survey- the largest pan-European study that provides internationally comparable data on a large number of health and social indicators. The present study included participants aged 65 years and older, and the analysis was conducted using several dimensions of psychosocial well-being and social support. An important aspect of the study was the gender-stratified analysis of

For peer review only

the association between psychosocial well-being and social support, which allowed a better understanding of the differences in the way men and women may respond to certain types of social support. Previous studies have underscored that gender-stratified analysis allows better insights into how biological, physiological, behavioural, environmental, and social factors interact differently in people of different genders.[28–30] As such, gender-stratified analysis is becoming increasingly important as researchers strive to improve the understanding of the unique healthcare needs of men and women.

This study has several important limitations to consider as well. Firstly, the data used in this analysis are cross-sectional and therefore no causal relationship can be established between the outcome and explanatory variables. Since the analysis was based on pre-existing data, the authors had no control over the selection and measurement of the study variables. As a result, not all dimensions of social support that are relevant to psychosocial well-being were possible to include in the analysis. Secondly, there was no specific information on what type of support was received by the participants from their contacts (e.g. financial, activities of daily living, emotional, healthcare visits) which could have produced a more detailed picture of the association between social support and psychosocial well-being. The analysis cannot clarify the nature or quality of the relationships as well. Someone receiving all the necessary support during the pandemic may experience higher health benefits compared to those receiving the support occasionally. The measures of psychosocial well-being are also self-reported and therefore remain subject to reporting bias. Future studies should focus on addressing these limitations and use more objective

For peer review only

indicators of social support and mental health. Regardless of these limitations, these findings make an important contribution to the literature on the positive role of social capital on psychosocial well-being. As the pandemic is starting to exert long-term social crises, which in turn are affecting people's health status, policymakers should attempt to make socially tailored decisions to minimize the effect of social isolation and provide support, especially for the most vulnerable communities.

## Conclusion

According to the findings, about one-third of the participants reported having nervousness, depression, loneliness, and sleep trouble. A quarter of them reported at least one, and about 8% reported having all of the four conditions, with the percentages being relatively higher among women than among men. A considerable inter-country difference was found in the corresponding percentages as well. Receiving help from own children, relatives, neighbours/friends/colleagues, and home care showed significantly lower risks of depression, nervousness, loneliness, and sleep trouble compared with those who didn't receive any help. Interestingly, the risk differences for all four indicators varied between men and women concerning the sources of social support such that the benefits are generally more pronounced among men than women.

**Author Contributions**: Conceptualization, L.J.; methodology, J.X., G.B., S.T., S.G.; software, J.X., G.B., S.T., S.G.; formal analysis, J.X., G.B., S.T., S.G.; data curation, G.B.; writing—original draft

For peer review only

preparation, L.J., J.X., G.B., S.T., S.G.; writing—review and editing L.J., J.X., G.B., S.T., S.G.; All authors have read and agreed to the published version of the manuscript.

**Funding**: This work was funded by grant 2022YFE0133000 from the National Key R&D Program of China, grant 72004073 from the National Natural Science Foundation of China, and grant 20YJC630134 from the Chinese Ministry of Education of Humanities and Social Science project. The funders had no role in the design or decision to publish the manuscript.

**Conflict of interest**: None to declare.

**Data availability**: Datasets are available to registered users through the SHARE website.

**Ethics Approval/Patient consent for publication**: Not applicable, data are secondary.

**Patient and public involvement**: No patient involved.

For peer review only

**References**

1. Dubey S, Biswas P, Ghosh R, Chatterjee S, Dubey MJ, Chatterjee S, et al. Psychosocial impact of COVID-19. Diabetes Metab Syndr. 2020;14:779–88.

2. Etowa J, Demeke J, Abrha G, Worku F, Ajiboye W, Beauchamp S, et al. Social determinants of the disproportionately higher rates of COVID-19 infection among African Caribbean and Black (ACB) population: A systematic review protocol. J Public Health Res. 2021;11:2274.

3. Hiscott J, Alexandridi M, Muscolini M, Tassone E, Palermo E, Soultsioti M, et al. The global impact of the coronavirus pandemic. Cytokine Growth Factor Rev. 2020;53:1–9.

4. Abdelghani M, Hassan MS, Elgohary HM, Fouad E. Exploring the factors associated with coronaphobia among physicians during the COVID-19 outbreak in Egypt. The Egyptian Journal of Neurology, Psychiatry and Neurosurgery. 2021;57.

5. Asmundson GJG, Taylor S. Coronaphobia: Fear and the 2019-nCoV outbreak. Journal of Anxiety Disorders. 2020;70:102196.

6. Hwang T-J, Rabheru K, Peisah C, Reichman W, Ikeda M. Loneliness and social isolation during the COVID-19 pandemic. Int Psychogeriatr. :1–4.

7. Brandt L, Liu S, Heim C, Heinz A. The effects of social isolation stress and discrimination on mental health. Transl Psychiatry. 2022;12:398.

8. McDaniels B, Subramanian I. Social isolation, loneliness and mental health sequelae of the Covid-19 pandemic in Parkinson's disease. Int Rev Neurobiol. 2022. https://doi.org/10.1016/bs.irn.2022.03.003.

9. Serafini G, Parmigiani B, Amerio A, Aguglia A, Sher L, Amore M. The psychological impact of COVID-19 on the mental health in the general population. QJM. 2020;113:531–7.

10. Why does COVID-19 disproportionately affect older people? - PMC. https://www.ncbi.nlm.nih.gov/pmc/articles/PMC7288963/. Accessed 19 Dec 2022.

11. Lowe C, Rafiq M, MacKay LJ, Letourneau N, Ng CF, Keown-Gerrard J, et al. Impact of the COVID-19 Pandemic on Canadian Social Connections: A Thematic Analysis. J Soc Pers Relat. 2022;:02654075221113365.

12. Impact of the COVID-19 Pandemic on Loneliness and Social Isolation: A Multi-Country Study - PMC. https://www.ncbi.nlm.nih.gov/pmc/articles/PMC8508181/.

For peer review only

13. McKinnon SA, Holloway BM, Santoro MS, May AC, Cronan TA. The Effects of Age, Mental Health, and Comorbidity on the Perceived Likelihood of Hiring a Healthcare Advocate. Calif J Health Promot. 2016;14:45–57.

14. Cosco TD, Randa C, Hopper S, Wagner KR, Pickering J, Best JR. Ageing and Mental Health in Canada: Perspectives from Law, Policy, and Longitudinal Research. J Popul Ageing. 2022;15:863–78.

15. Rosso AL, Taylor JA, Tabb LP, Michael YL. MOBILITY, DISABILITY, AND SOCIAL ENGAGEMENT IN OLDER ADULTS. J Aging Health. 2013;25:617–37.

16. Sayin Kasar K, Karaman E. Life in lockdown: Social isolation, loneliness and quality of life in the elderly during the COVID-19 pandemic: A scoping review. Geriatr Nurs. 2021;42:1222–9.

17. Harandi TF, Taghinasab MM, Nayeri TD. The correlation of social support with mental health: A meta-analysis. Electron Physician. 2017;9:5212–22.

18. Li F, Luo S, Mu W, Li Y, Ye L, Zheng X, et al. Effects of sources of social support and resilience on the mental health of different age groups during the COVID-19 pandemic. BMC Psychiatry. 2021;21:16.

19. Roohafza HR, Afshar H, Keshteli AH, Mohammadi N, Feizi A, Taslimi M, et al. What's the role of perceived social support and coping styles in depression and anxiety? J Res Med Sci. 2014;19:944–9.

20. Scardera S, Perret LC, Ouellet-Morin I, Gariépy G, Juster R-P, Boivin M, et al. Association of Social Support During Adolescence With Depression, Anxiety, and Suicidal Ideation in Young Adults. JAMA Netw Open. 2020;3:e2027491.

21. Börsch-Supan A. Survey of Health, Ageing and Retirement in Europe (SHARE) Wave 9. COVID-19 Survey 2. 2022.

For peer review only

22. Börsch-Supan A, Brandt M, Hunkler C, Kneip T, Korbmacher J, Malter F, et al. Data Resource Profile: the Survey of Health, Ageing and Retirement in Europe (SHARE). Int J Epidemiol. 2013;42:992–1001.

23. Kim H-Y. Statistical notes for clinical researchers: Risk difference, risk ratio, and odds ratio. Restor Dent Endod. 2017;42:72–6.

24. Riecher-Rössler A. Sex and gender differences in mental disorders. The Lancet Psychiatry. 2017;4:8–9.

25. McLean CP, Asnaani A, Litz BT, Hofmann SG. Gender Differences in Anxiety Disorders: Prevalence, Course of Illness, Comorbidity and Burden of Illness. J Psychiatr Res. 2011;45:1027–35.

26. McNab S, Fisher J, Honikman S, Muvhu L, Levine R, Chorwe-Sungani G, et al. Comment: silent burden no more: a global call to action to prioritize perinatal mental health. BMC Pregnancy and Childbirth. 2022;22:308.

27. Grey I, Arora T, Thomas J, Saneh A, Tohme P, Abi-Habib R. The role of perceived social support on depression and sleep during the COVID-19 pandemic. Psychiatry Res. 2020;293:113452.

28. Miani C, Wandschneider L, Niemann J, Batram-Zantvoort S, Razum O. Measurement of gender as a social determinant of health in epidemiology—A scoping review. PLoS One. 2021;16:e0259223.

29. Bolte G, Jacke K, Groth K, Kraus U, Dandolo L, Fiedel L, et al. Integrating Sex/Gender into Environmental Health Research: Development of a Conceptual Framework. Int J Environ Res Public Health. 2021;18:12118.

30. Short SE, Yang YC, Jenkins TM. Sex, Gender, Genetics, and Health. Am J Public Health. 2013;103 Suppl 1:S93–101.

For peer review only

For peer review only

Figure legends:

**Figure 1: Percentage of participants who reported having depression, Nervousness, Loneliness, and Sleep Trouble by country.**

As shown in figure 1, the percentage of self-reported depression was over 40% in Portugal (46.44%), Bulgaria (42.5%), and Hungary (42.33%). Portugal (53.37%), Bulgaria (50.83%) also had the highest percentages of self-reported nervousness. The percentage of loneliness was highest in Greece (52.91%) and that of sleep trouble was highest in Estonia (49.13%).

**Figure 2: comparison of the total number of conditions reported by male and female participants.**

Figure 2 shows that about a quarter of the men and women reported at least one of the four conditions. However, the percentage of reporting more than one condition was higher among

For peer review only

women than men. For example, 10.72% of the women reported having all four conditions compared with 4.81% among men.

For peer review only

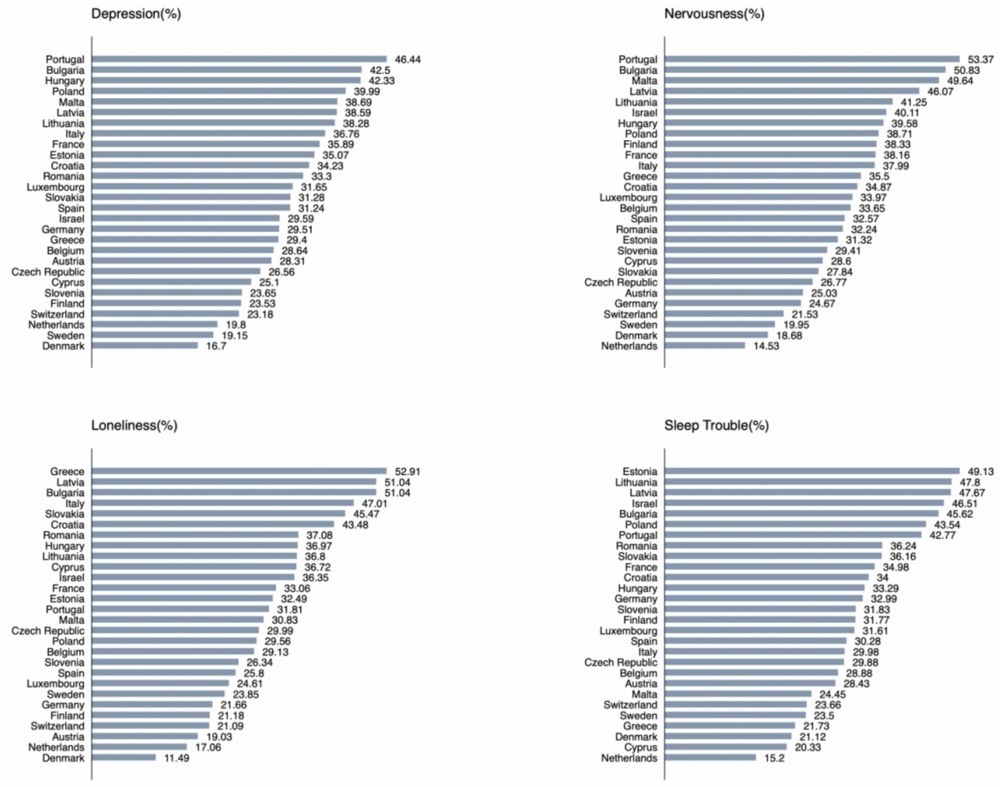


Percentage of participants who reported having depression,Nervousness, Loneliness, and Sleep Trouble by country.

165x129mm (220 x 220 DPI)

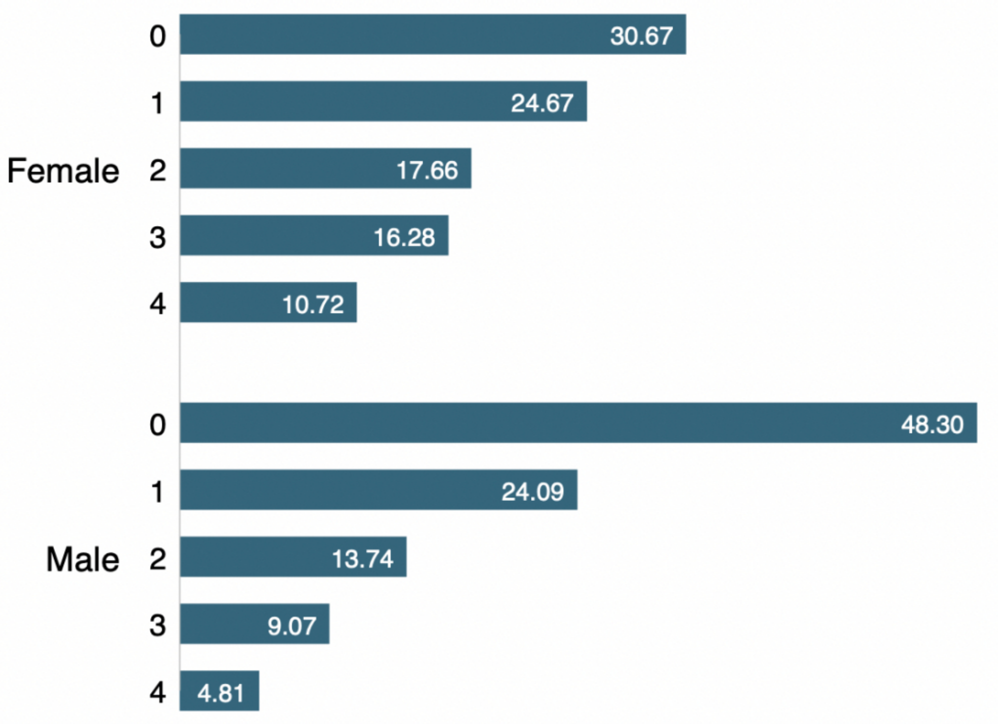


comparison of the total number of conditions reported by male and female participants.

118x86mm (220 x 220 DPI)

Appendix

Table 1a: Sample description (Share 8).

| age | Freq. | Percent | Cum. |
|---|---|---|---|
| 65-69 years | 9857 | 26.92 | 26.92 |
| 70-74 years | 9719 | 26.54 | 53.46 |
| 75-79 years | 7508 | 20.50 | 73.96 |
| 80-84 years | 5399 | 14.74 | 88.70 |
| 85+ | 4138 | 11.30 | 100.00 |
| | | | |
| Sex | Freq. | Percent | Cum. |
| Male | 15719 | 42.92 | 42.92 |
| Female | 20902 | 57.08 | 100.00 |
| | | | |
| Employment status | Freq. | Percent | Cum. |
| Retired | 32195 | 88.03 | 88.03 |
| Employment | 1626 | 4.45 | 92.48 |

For peer review only

| | | | |
|---|---|---|---|
| Unemployed | 79 | 0.22 | 92.69 |
| Permanently sick or disabled | 298 | 0.81 | 93.51 |
| Homemaker | 1980 | 5.41 | 98.92 |
| Other | 395 | 1.08 | 100.00 |
| | | | |
| Rating of subjective health | Freq. | Percent | Cum. |
| Excellent | 1266 | 3.46 | 3.46 |
| Very good | 4789 | 13.09 | 16.55 |
| Good | 14290 | 39.05 | 55.60 |
| Fair | 12106 | 33.08 | 88.69 |
| Poor | 4140 | 11.31 | 100.00 |
| | | | |
| Country | | | |
| Austria | 1943 | 5.31 | 5.31 |
| Germany | 1466 | 4.00 | 9.31 |
| Sweden | 856 | 2.34 | 11.65 |
| Netherlands | 592 | 1.62 | 13.26 |
| Spain | 1545 | 4.22 | 17.48 |
| Italy | 2528 | 6.90 | 24.38 |
| France | 1458 | 3.98 | 28.37 |

For peer review only

| | | | |
|---|---|---|---|
| Denmark | 1143 | 3.12 | 31.49 |
| Greece | 2515 | 6.87 | 38.36 |
| Switzerland | 1452 | 3.96 | 42.32 |
| Belgium | 2427 | 6.63 | 48.95 |
| Israel | 1132 | 3.09 | 52.04 |
| Czech Republic | 1808 | 4.94 | 56.98 |
| Poland | 1795 | 4.90 | 61.88 |
| Luxembourg | 581 | 1.59 | 63.46 |
| Hungary | 715 | 1.95 | 65.42 |
| Portugal | 860 | 2.35 | 67.76 |
| Slovenia | 2313 | 6.32 | 74.08 |
| Estonia | 3012 | 8.22 | 82.31 |
| Croatia | 1305 | 3.56 | 85.87 |
| Lithuania | 774 | 2.11 | 87.98 |
| Bulgaria | 480 | 1.31 | 89.29 |
| Cyprus | 490 | 1.34 | 90.63 |
| Finland | 855 | 2.33 | 92.97 |
| Latvia | 623 | 1.70 | 94.67 |
| Malta | 548 | 1.50 | 96.16 |
| Romania | 919 | 2.51 | 98.67 |

For peer review only

| | | | |
|---|---|---|---|
| Slovakia | 486 | 1.33 | 100.00 |

For peer review only

BMJ Open

# Social support and psychosocial well-being among older adults in Europe during the COVID-19 pandemic: a cross-sectional study



| | |
|---|---|
| Journal: | *BMJ Open* |
| Manuscript ID | bmjopen-2022-071533.R1 |
| Article Type: | Original research |
| Date Submitted by the Author: | 02-Jun-2023 |





| | |
|---|---|
| Complete List of Authors: | Lu, Ji; Tongji Medical College,<br>Xiong, Juyang; Huazhong University of Science and Technology Tongji Medical College, School of Medicine and Health Management<br>Tang, Shangfeng; Tongji Medical College of Huazhong University of Science and Technology, Health mangament<br>Bishwajit, Ghose; University of Ottawa, Faculty of Health Sciences;<br>Guo, Shuyan; National Institute of Hospital Administration |





| | |
|---|---|
| <b>Primary Subject Heading</b>: | Mental health |
| Secondary Subject Heading: | Epidemiology |
| Keywords: | COVID-19, MENTAL HEALTH, SOCIAL MEDICINE |



SCHOLARONE™
Manuscripts

*I, the Submitting Author has the right to grant and does grant on behalf of all authors of the Work (as defined in the below author licence), an exclusive licence and/or a non-exclusive licence for contributions from authors who are: i) UK Crown employees; ii) where BMJ has agreed a CC-BY licence shall apply, and/or iii) in accordance with the terms applicable for US Federal Government officers or employees acting as part of their official duties; on a worldwide, perpetual, irrevocable, royalty-free basis to BMJ Publishing Group Ltd ("BMJ") its licensees and where the relevant Journal is co-owned by BMJ to the co-owners of the Journal, to publish the Work in this journal and any other BMJ products and to exploit all rights, as set out in our licence.*

*The Submitting Author accepts and understands that any supply made under these terms is made by BMJ to the Submitting Author unless you are acting as an employee on behalf of your employer or a postgraduate student of an affiliated institution which is paying any applicable article publishing charge ("APC") for Open Access articles. Where the Submitting Author wishes to make the Work available on an Open Access basis (and intends to pay the relevant APC), the terms of reuse of such Open Access shall be governed by a Creative Commons licence – details of these licences and which Creative Commons licence will apply to this Work are set out in our licence referred to above.*

*Other than as permitted in any relevant BMJ Author's Self Archiving Policies, I confirm this Work has not been accepted for publication elsewhere, is not being considered for publication elsewhere and does not duplicate material already published. I confirm all authors consent to publication of this Work and authorise the granting of this licence.*

For peer review only

# Social support and psychosocial well-being among older adults in Europe during the COVID-19 pandemic: a cross-sectional study

**Lu Ji[1*], PhD; Juyang Xiong[1*], PhD; Shangfeng Tang[1], PhD; Ghose Bishwajit[2], PhD; Shuyan Guo[3]**

1 Lu Ji， School of Medicine and Health Management, Tongji Medical College of Huazhong University of Science and Technology, Wuhan, China. Jilu2020@hust.edu.cn

1 Juyang Xiong, School of Medicine and Health Management, Tongji Medical College of Huazhong University of Science and Technology, Wuhan, China. xiongjuyang@hust.edu.cn

1 Shangfeng Tang, School of Medicine and Health Management, Tongji Medical College of Huazhong University of Science and Technology, Wuhan, China. Sftang2018@hust.edu.cn

2 Ghose Bishwajit, Faculty of Health Sciences, University of Ottawa, Ottawa, Ontario, Canada. Brammaputram@gmail.com

3 Shuyan Guo, National Institute of Hospital Administration, National Health Commission, Beijing, China, guoshuyan@niha.org.cn

*Lu Ji and Juyang Xiong contributed equally

Correspondence to Shuyan Guo, guoshuyan@niha.org.cn

Word count: 3800.

For peer review only

**Abstract**: The objective of the study was to identify the association between social support and psychosocial well-being among men and women aged over 65 years in Europe during the COVID-19 pandemic.

**Methods**: Cross-sectional data on 36,621 men (n=15,719) and women (n=20,902) aged 65 years or higher were obtained from the ninth round of the Survey of Health, Ageing and Retirement in Europe (SHARE). The outcomes were measured by psychosocial well-being reflected with self-reported depression, nervousness, loneliness and sleep disturbances. Social support was measured in terms of receiving help from own children, relatives, and neighbours/friends/colleagues since the pandemic outbreak.

**Result**: About one-third of the participants reported depression (31.03%), nervousness (32.85%), loneliness (32.23%), and sleep trouble (33.01%). The results of multivariable regression analysis revealed that social support was a protective factor to psychological well-being. For instance, receiving help from own children [RD= -0.13, 95% CI = -0.14, -0.12], relatives [RD= -0.08, 95% CI = -0.11, -0.06], neighbours/friends/colleagues [RD= -0.11, 95% CI = -0.13, -0.09], and receiving home care [RD= -0.20, 95% CI = -0.22, -0.18] showed significantly lower risk difference for depression. Similar findings were noted for loneliness, nervousness, and sleep trouble as well, with the risk difference being slightly different for men and women in the gender-stratified analysis. For instance, the risk difference in depression for receiving help from own children was -0.10 [95% confidence interval = -0.12, -0.08] among men compared with -0.12 [95% confidence interval = -0.14, -0.11] among women. The risk differences in the outcome measures were calculated using Generalized Linear Model for binomial family.

For peer review only

**Conclusion**: Findings of the present study highlight a protective role of social support on psychological well-being among both men and women. Developing strategies to promote social support, especially among older adults, may mitigate the rising burden of psychological illness during the COVID-19 pandemic.

**Keywords**: COVID-19; aging health; Mental health; Social support; Well-being.

## Strengths and limitations

•This study focused on the association between social support and psychosocial well-being among older adults during the COVID-19 pandemic in EU countries.

•The analysis conducted using multiple dimensions of psychosocial well-being and social support.

• The cross-sectional nature of the surveys prevents establishing causal relationships between outcome and explanatory variables.

• Inclusion of all relevant sociocultural factors was not possible due to the secondary analysis.

## Introduction

The COVID-19 pandemic has become one of the most devastating health and social crises in generations due to its long-term impacts on physical and mental health, social life and livelihoods

For peer review only

worldwide.[1–3] A pervasive crisis is the emergence of a phenomenon known as "coronaphobia" - fear or anxiety related to the virus and its potential impacts on the psychosocial well-being of individuals and communities at large.[4,5] A growing number of studies have highlighted the prevalence of mental illness in the context of lifestyle such as higher consumption of psychoactive drugs and widespread social isolation and loneliness.[6–9] The fear of contracting the virus has compelled people to stay at home for an extended period of time, particularly affecting elderly individuals who face an elevated risk of severe illness from the virus.[10]

For peer review only

The measures to control the COVID-19 pandemic, such as physical distancing, have caused disruptions to a healthy and active lifestyle, as well as the opportunities to maintain social connections and seek help in times of need.[11] The impact of social isolation and loneliness on mental health has been an increasing concern in the wake of the global pandemic, as socialization opportunities have become limited and physical distancing rules have been put in place.[12] As such, there has been a dramatic decrease in social interaction and support, which is particularly concerning for peoples' psychosocial well-being during the global health crisis. Prolonged isolation can lead to feelings of loneliness, depression and anxiety and can take a toll on mental health of older adults due to their higher dependence on physical and emotional care.

The feeling of depression and loneliness are common mental health concerns that can affect people from all walks of life, especially among older adults. This is due to a range of factors, including changes in physical health and lifestyle, increased isolation and loneliness, financial concerns, and difficulty in adapting to the technological advances of modern life. The COVID-19 pandemic has fuelled social isolation and loneliness mainly through fewer opportunities for meeting friends and families, organizing or participating in social gatherings and reduced social support.[13] Social support, which is a key aspect of social capital, has been shown to be strongly correlated with mental health in previous studies.[14,15] Perceived social support refers to the

For peer review only

subjective availability of psychological, physical, and financial help from social relationships such as family, friends, neighbours, and community members that is available in times of need. Social support plays an important role in mitigating the impact of social isolation and can help reduce stress, improve mood, and promote positive coping mechanisms.[16] Social support also provides a sense of belonging and connection, which can offset feelings of loneliness and isolation. Several previous studies have demonstrated the link between social relationships and support and mental health.[14,16,17] However, there is not enough evidence regarding this relationship among older adults in European countries in the context of the COVID-19 pandemic. The present study therefore investigates the relationship between social support and psychosocial well-being among older adults in Europe using the data from the Survey of Health Ageing, and Retirement (WAVE 9).

## Methods

### Data source

Data for this study were derived from the ninth wave of the Survey of Health Ageing, and Retirement in Europe (SHARE).[18] The survey aims to provide a framework for researchers to better understand population ageing and covers most of the European Union and Israel. SHARE has been adopted as a model by a number of ageing surveys throughout the world and is harmonized with the US Health and Retirement Study (HRS) and the English Longitudinal Study of Ageing (ELSA).[19] The SHARE survey collects data on a diverse range of health and socioeconomic indicators among individuals including demographics, living conditions, income and employment status, mental health, satisfaction with health conditions and social networks. The

For peer review only

ninth wave of the Survey included questions relevant to COVID-19, such as receiving support from social connections to obtain basic necessities. Data for this survey were collected via computer-assisted telephone interviews between June and August of 2021 among individuals aged 50 or older. For the purpose of the present study, the analysis was limited to participants aged 65 years or older only.

**Description of the variables**

The outcome measures included four indicators of psychosocial well-being assessed by self-reported nervousness, depression, sleep disturbances and loneliness. These indicators were assessed by the following questions: 1) felt nervous in last month (Yes/No), 2) sad or depressed in last month (Yes/No), 3) trouble sleeping recently (Yes/No), 4) how often feelings of loneliness (Often/Some of the times/Hardly ever or never). For loneliness, the answers were recoded to 'Yes' for responses 'Often/Some of the times' and to 'No' for responses 'Hardly ever or never'. Social support was assessed in terms of receiving help in obtaining necessities during the pandemic from own children, relatives, and neighbours/friends/colleagues to obtain necessities since the outbreak. These indicators were assessed by the following questions: 1) help received from own children to obtain necessities since the outbreak (Yes/No), 2) help received from other relatives to obtain necessities since the outbreak (Yes/No), 3) help received from neighbours/friends/colleagues to obtain necessities (Yes/No), 4) received regular home care during last 3 months (Yes/No). Apart from the indicators of social support, the analysis also included several socioeconomic variables based on their known association with mental health status such as age (65-69 years/ 70-74 years/

For peer review only

75-79 years/ 80-84 years/ 85+ years), sex (male/female), employment situation (Retired/ Employed or self-employed/ Unemployed/ Permanently sick or disabled? Homemaker/ Other), and country of residence (list of countries provided in appendix).

## Statistical Analysis

All statistical analyses were conducted using Stata 17 (Stata Corp., College Station, Texas). The dataset was first examined to make sure the sample population was defined correctly (65 years and older) and there were no missing values and outliers. Descriptive statistics were carried out to calculate the percentage of participants reporting nervousness, depression, loneliness, and sleep trouble. Sex and country-level percentages were presented as bar charts. In the next step, we conducted generalized linear models (GLMs) for binomial family to measure the risk difference (with 95% confidence intervals) in the four outcome variables (nervousness, depression, loneliness, and sleep trouble). The results of GLMs were presented as risk difference (RD), defined as the risk of a condition between an exposed group and an unexposed group.[20] A positive RD value indicates a higher risk by the exposure, whereas a negative one indicates a lower risk. Given the well-documented gender differences in mental health outcomes, the analysis was stratified by male and female participants for each outcome variable. The level of statistical significance for all associations was set at 0.05.

**Patient and public involvement**: No patient involved.

For peer review only

**Results**

The sociodemographic characteristics of the sample population are available in the appendix file. A greater percentage of the participants were in the age bracket of 65-69 years (n= 26.92%), female (n= 57.08%), retired (n= 88.03%), and rated general health status as good (n= 39.05%).

Sample size for the participating countries was as follows: Austria (n= 1943), Germany (n= 1466), Sweden (n= 856), Netherlands (n= 592), Spain (n= 1545), Italy (n= 2528), France (n= 1458), Denmark (n= 1143), Greece (n= 2515), Switzerland (n= 1452), Belgium (n= 2427), Israel (n= 1132), Czech Republic (n= 1808), Poland (n= 1795), Luxembourg (n= 581), Hungary (n= 715), Portugal (n= 860), Slovenia (n= 2313), Estonia (n= 3012), Croatia (n= 1305), Lithuania (n= 774), Bulgaria (n= 480), Cyprus (n= 490), Finland (n= 855), Latvia (n= 623), Malta (n= 548), Romania (n= 919), Slovakia (n= 486).

Among the four psychosocial well-being indicators, sleep trouble (33.01%) was the most commonly reported one (table 1), followed by nervousness (32.85%) and loneliness (32.23%). As described in table 1, the percentage of reporting depression (77.08% vs 62.88%), nervousness (73.56% vs 62.33%), and loneliness (76.43% vs 61.27%) was higher in men, while that of sleep trouble was higher in women (25.73% vs 38.49%).

For peer review only

**Table 1: Percentage of participants who reported having depression, Nervousness, Loneliness, and Sleep Trouble by sex.**

| | Percentage of Total | Percentage of total Men | Percentage of total Women |
|---|---|---|---|
| Depression | 31.03 | 22.92 | 37.12 |
| Nervousness | 32.85 | 26.44 | 37.67 |
| Loneliness | 32.23 | 23.57 | 38.73 |
| Sleep trouble | 33.01 | 25.73 | 38.49 |

**Figure 1: Percentage of participants who reported having depression, Nervousness, Loneliness, and Sleep Trouble by country.**

For peer review only

**Figure 2: comparison of the total number of conditions reported by male and female participants.**

The results of multivariable regression analysis on the association between self-reported depression and social support are shown in table 2. Receiving help from own children [RD= -0.13, 95% confidence interval = -0.14, -0.12], relatives [RD= -0.08, 95% confidence interval = -0.11, -0.06], neighbours/friends/colleagues [RD= -0.11, 95% confidence interval = -0.13, -0.09], and receiving home care [RD= -0.20, 95% confidence interval = -0.22, -0.18] showed significantly lower risk difference for depression, with the differences being slightly higher among women compared with men. For instance, the risk difference in depression for receiving help from own children was -0.10 [95% confidence interval = -0.12, -0.08] among men compared with -0.12 [95% confidence interval = -0.14, -0.11] among women.

Table 2: Association between social support and self-reported depression.

For peer review only

| | Full sample | Men | Women |
|---|---|---|---|
| | | | |
| **Received help from own children (Yes)** | ref | ref | ref |
| No | -0.13*** [-0.14,-0.12] | -0.10*** [-0.12,-0.08] | -0.12*** [-0.14,-0.11] |
| **Received help from relatives (Yes)** | ref | ref | ref |
| No | -0.08*** [-0.11,-0.06] | -0.07*** [-0.11,-0.03] | -0.08*** [-0.11,-0.05] |
| **Received help from neighbors/friends/colleagues (yes)** | ref | ref | ref |
| No | -0.11*** [-0.13,-0.09] | -0.09*** [-0.12,-0.06] | -0.11*** [-0.13,-0.08] |
| **Received home care (Yes)** | ref | ref | ref |
| No | -0.20*** [-0.22,-0.18] | -0.19*** [-0.22,-0.15] | -0.19*** [-0.21,-0.16] |

N.B. cells represent risk differences with 95% confidence intervals. All models are adjusted for age, sex, employment status, subjective health status, and country of residence. Level of significance: * $p < 0.05$, ** $p < 0.01$, *** $p < 1$

The results of multivariable regression analysis on the association between social support and self-reported nervousness are shown in table 3. Receiving help from own children [RD= -0.10, 95% confidence interval = -0.12, -0.09], relatives [RD= -0.05, 95% confidence interval = -0.07, -0.03], neighbours/friends/colleagues [RD= -0.08, 95% confidence interval = -0.10, -0.06], and

For peer review only

receiving home care [RD= -0.15, 95% confidence interval = -0.17, -0.13] showed significantly lower risk difference for nervousness. In the gender-stratified analysis, receiving help from own children showed a relatively higher risk difference among women, whereas receiving help from neighbours/friends/colleagues showed a relatively higher risk difference among men.

For peer review only

**Table 3:** Association between social support and self-reported nervousness.

| | **Full sample** | Men | Women |
|---|---|---|---|
| **Received help from own children (Yes)** | ref | ref | ref |
| No | $-0.10^{***}$ [-0.12,-0.09] | $-0.10^{***}$ [-0.11,-0.08] | $-0.09^{***}$ [-0.11,-0.08] |
| **Received help from relatives (Yes)** | ref | ref | ref |
| No | $-0.05^{***}$ [-0.07,-0.03] | $-0.04^{*}$ [-0.08,-0.01] | $-0.05^{***}$ [-0.08,-0.02] |
| **Received help from neighbors/friends/colleagues (yes)** | ref | ref | ref |
| No | $-0.08^{***}$ [-0.10,-0.06] | $-0.06^{***}$ [-0.09,-0.02] | $-0.09^{***}$ [-0.11,-0.06] |
| **Received home care (Yes)** | ref | ref | ref |
| No | $-0.15^{***}$ [-0.17,-0.13] | $-0.15^{***}$ [-0.18,-0.11] | $-0.15^{***}$ [-0.17,-0.12] |

N.B. cells represent risk differences with 95% confidence intervals. All models are adjusted for age, sex, employment status, subjective health status, and country of residence. Level of significance: $^{*}$ $p < 0.05$, $^{**}$ $p < 0.01$, $^{***}$ $p < 1$

The results of multivariable regression analysis on the association between social support and loneliness are shown in table 4. Receiving help from own children [RD= -0.13, 95% confidence interval = -0.14, -0.11] relatives [RD= -0.08, 95% confidence interval = -0.10,-0.05] neighbours/friends/colleagues[RD= -0.12, 95% confidence interval = -0.13, -0.10], and receiving home care [RD= -0.12, 95% confidence interval = -0.14,-0.10] showed significantly lower risk difference for loneliness. The risk difference was relatively higher among men who reported receiving help from own children, relatives, and neighbours/friends/colleagues.

**Table 4:** Association between social support and self-reported loneliness.

| | Full sample | Men | Women |
|---|---|---|---|
| **Received help from own children (Yes)** | ref | ref | ref |
| No | -0.13*** [-0.14,-0.11] | -0.09*** [-0.11,-0.08] | -0.12*** [-0.13,-0.10] |
| **Received help from relatives (Yes)** | ref | ref | ref |
| No | -0.08*** [-0.10,-0.05] | -0.07*** [-0.11,-0.03] | -0.08*** [-0.10,-0.05] |
| | | | |
| **Received help from neighbors/friends/colleagues (yes)** | ref | ref | ref |

For peer review only

| | | | |
|---|---|---|---|
| No | -0.12*** [-0.13,-0.10] | -0.07*** [-0.10,-0.04] | -0.12*** [-0.15,-0.10] |
| **Received home care (Yes)** | ref | ref | ref |
| No | -0.12*** [-0.14,-0.10] | -0.13*** [-0.16,-0.10] | -0.10*** [-0.12,-0.07] |

N.B. cells represent risk differences with 95% confidence intervals. All models are adjusted for age, sex, employment status, subjective health status, and country of residence. Level of significance: * $p < 0.05$, ** $p < 0.01$, *** $p < 1$

The results of multivariable regression analysis on the association between social support and sleep trouble are shown in table 5. Receiving help from own children [RD= -0.10, 95% confidence interval = -0.11,-0.09], relatives [RD= -0.05, 95% confidence interval = -0.08, -0.03], neighbours/friends/colleagues [-0.07, 95% confidence interval = -0.09,-0.05], and receiving home care [RD= -0.12, 95% confidence interval =-0.14,-0.10] showed significantly lower risk difference for sleep trouble. In the gender-stratified analysis, receiving help from own children and from neighbours/friends/colleagues showed a relatively higher risk difference among men.

For peer review only

**Table 5:** Association between social support and sleep trouble.

| | Full sample | Men | Women |
|---|---|---|---|
| **Received help from own children (Yes)** | ref | ref | ref |
| No | $-0.10^{***}$ [-0.11,-0.09] | $-0.07^{***}$ [-0.09,-0.05] | $-0.09^{***}$ [-0.10,-0.08] |
| | | | |
| **Received help from relatives (Yes)** | ref | ref | ref |
| No | $-0.05^{***}$ [-0.08,-0.03] | $-0.06^{**}$ [-0.10,-0.02] | $-0.04^{**}$ [-0.07,-0.01] |
| **Received help from neighbors/friends/colleagues (yes)** | ref | ref | ref |
| No | $-0.07^{***}$ [-0.09,-0.05] | $-0.06^{***}$ [-0.09,-0.03] | $-0.07^{***}$ [-0.09,-0.04] |
| **Received home care (Yes)** | ref | ref | ref |
| No | $-0.12^{***}$ [-0.14,-0.10] | $-0.13^{***}$ [-0.16,-0.10] | $-0.10^{***}$ [-0.12,-0.07] |

N.B. cells represent risk differences with 95% confidence intervals. All models are adjusted for age, sex, employment status, subjective health status, and country of residence. Level of significance: $^{*}$ $p < 0.05$, $^{**}$ $p < 0.01$, $^{***}$ $p < 1$

**Discussion**

Given the rising burden of mental illness during the COVID-19 pandemic, it's important to explore the potential role of the social determinants that can affect mental health, especially among



For peer review only

older adults who share a greater vulnerability to both COVID-19 and mental health conditions. The present study aimed to assess the relationship between social support and indicators of psychosocial well-being among older men and women in 27 countries in Europe and Israel using data from the Survey of Health Ageing, and Retirement. Our findings suggest that about one-third of the participants reported depression, nervousness, loneliness, and sleep trouble, with the percentage of all four conditions being relatively higher among women. Overall, about a quarter of the participants reported at least one, and about 8% reported having all of the four conditions. However, the percentage of reporting multiple conditions was higher among women than among men, indicating a greater burden of psychological issues among women. Previous studies have reported a higher burden of mental health issues among women and attempted to explain the gender differences in mental disorders through various biological (pregnancy, hormonal) and psychosocial factors (self-esteem, social empowerment).[21–23] Apart from the gender differences, we observed remarkable inter-country differences in the corresponding percentages as well. For instance, the percentage of depression ranged from 46.44% in Portugal to 16.7% in Denmark, nervousness from 53.37% in Portugal to 14.53% in the Netherlands, loneliness from 52.91% in Greece to 11.49% in Denmark, and sleep trouble from 49.13% in Estonia to 15.2% in the Netherlands. The country-level disparities might be rooted in the health policies related to the pandemic and health and social conditions that are relevant to mental health in individual countries that should be investigated through more in-depth studies.

For peer review only

Regarding the association between social support in obtaining the necessities during the pandemic and the outcome measures, receiving help from own children, relatives, neighbours/friends/colleagues, and home care showed significantly lower risks of depression, nervousness, loneliness, and sleep trouble compared with those didn't receive any help. Several previous studies have reported the positive association between social support and psychosocial well-being through a social capital lens. According to a cohort study conducted in Quebec, Canada, adolescents who felt they had more social support one year later reported having fewer mental health issues, concluding that perceived social support, especially in those who have mental health issues in youth, may shield against mental health issues as people enter adulthood.[17] A meta-analysis involving 64 studies reported a mean effect size of 0.356, indicating a moderate effect size of social support on mental health.[14] Another study conducted in Lebanon using structured questionnaires for depression (Patient Health Questionnaire), loneliness (UCLA Loneliness Scale) and social support (Multidimensional Scale of Perceived Social Support) found that the rates of depression and loneliness were significantly higher among participants who experienced self-isolation compared to those who didn't.[24] The same study reported that the risk of depressive symptoms and poor sleep quality were respectively 63% and 52% lower among individuals who reported higher levels of social support compared to those with low perceived social support.[24] In the context of the COVID-19 pandemic, receiving social support was reported to be inversely associated with mental health problems in several countries including China,[25] Spain,[26] Poland and Ukraine.[27]

For peer review only

The findings of the current study are consistent with previous ones regarding the positive role of social support on psychological well-being. Social support can take many forms, from family and friends to community groups and online forums, and serves the purpose of providing a sense of connection and belonging and improving coping skills, which can be vital for people who are feeling isolated. Lack of social support can therefore exacerbate the effects of social isolation and lead to further mental health problems. Arguably, the social stressors associated with the pandemic can lead to short- and long-term psychological issues such as anxiety and depression, which in turn can contribute to feelings of loneliness and isolation. With the increasing demands of modern life, individuals are becoming more aware of the protective effects of relationships on health and overall quality of life as it can act as a buffer against social stressors and promote positive mental well-being. Mental health experts have also alarmed about the dangers of social isolation since the start of the COVID-19 pandemic. Policymakers and social researchers should therefore aim to develop innovative strategies for promoting this invaluable resource to build more resilient communities that can help mitigate the long-term mental health impacts of the pandemic.

As far as we are concerned, this is the first study to focus on the association between social support and psychosocial well-being among the older adults during the COVID-19 pandemic in EU countries. The sample size was large and the data were obtained from a reputed population survey- the largest pan-European study that provides internationally comparable data on a large number of health and social indicators. The present study included participants aged 65 years and

For peer review only

older, and the analysis was conducted using several dimensions of psychosocial well-being and social support. An important aspect of the study was the gender-stratified analysis of the association between psychosocial well-being and social support, which allowed a better understanding of the differences in the way men and women may respond to certain types of social support. Previous studies have underscored that gender-stratified analysis allows better insights into how biological, physiological, behavioural, environmental, and social factors interact differently in people of different genders.[28–30] As such, gender-stratified analysis is becoming increasingly important as researchers strive to improve the understanding of the unique healthcare needs of men and women. The importance of social support for psychological well-being is a well-established finding in previous literature. However, in the present study, it was not possible to directly compare this phenomenon with the pre-pandemic time due to the unavailability of similar data. This study has several important limitations to consider as well. Firstly, the data used in this analysis are cross-sectional and therefore no causal relationship can be established between the outcome and explanatory variables. Since the analysis was based on pre-existing data, the authors had no control over the selection and measurement of the study variables. As a result, not all dimensions of social support that are relevant to psychosocial well-being were possible to include in the analysis. Secondly, there was no specific information on what type of support was received by the participants from their contacts (e.g. financial, activities of daily living, emotional, healthcare visits) which could have produced a more detailed picture of the association between social support and psychosocial well-being. The analysis cannot clarify the nature or quality of the relationships as well. Someone receiving all the necessary support during the pandemic may

For peer review only

experience higher health benefits compared to those receiving the support occasionally. The measures of psychosocial well-being are also self-reported and therefore remain subject to reporting bias. Future studies should focus on addressing these limitations and use more objective indicators of social support and mental health. Regardless of these limitations, these findings make an important contribution to the literature on the positive role of social capital on psychosocial well-being. As the pandemic is starting to exert long-term social crises, which in turn are affecting people's health status, policymakers should attempt to make socially tailored decisions to minimize the effect of social isolation and provide support, especially for the most vulnerable communities.

**Conclusion**

According to the findings, about one-third of the participants reported having nervousness, depression, loneliness, and sleep trouble. A quarter of them reported at least one, and about 8% reported having all of the four conditions, with the percentages being relatively higher among women than among men. A considerable inter-country difference was found in the corresponding percentages as well. Receiving help from own children, relatives, neighbours/friends/colleagues, and home care showed significantly lower risks of depression, nervousness, loneliness, and sleep trouble compared with those who didn’t receive any help. Interestingly, the risk differences for all four indicators varied between men and women concerning the sources of social support such that the benefits are generally more pronounced among men than women.

For peer review only

**Author Contributions**: Conceptualization, L.J.; methodology, J.X., G.B., S.T., S.G.; software, J.X., G.B., S.T., S.G.; formal analysis, J.X., G.B., S.T., S.G.; data curation, G.B.; writing—original draft preparation, L.J., J.X., G.B., S.T., S.G.; writing—review and editing L.J., J.X., G.B., S.T., S.G.; All authors have read and agreed to the published version of the manuscript.

**Funding**: This work was funded by grant 2022YFE0133000 from the National Key R&D Program of China, grant 72004073 from the National Natural Science Foundation of China, and grant 20YJC630134 from the Chinese Ministry of Education of Humanities and Social Science project. The funders had no role in the design or decision to publish the manuscript.

**Conflict of interest**: None to declare.

**Data availability**: Survey of Health, Ageing and Retirement in Europe. DOI: 10.6103/SHARE.w9ca.800. 2022-02-10

**Ethics Approval/Patient consent for publication**: Not applicable, data are secondary.

For peer review only

**References**

1. Dubey, S. *et al.* Psychosocial impact of COVID-19. *Diabetes Metab Syndr* **14**, 779–788 (2020).

2. Etowa, J. *et al.* Social determinants of the disproportionately higher rates of COVID-19 infection among African Caribbean and Black (ACB) population: A systematic review protocol. *J Public Health Res* **11**, 2274 (2021).

3. Hiscott, J. *et al.* The global impact of the coronavirus pandemic. *Cytokine Growth Factor Rev* **53**, 1–9 (2020).

4. Abdelghani, M., Hassan, M. S., Elgohary, H. M. & Fouad, E. Exploring the factors associated with coronaphobia among physicians during the COVID-19 outbreak in Egypt. *The Egyptian Journal of Neurology, Psychiatry and Neurosurgery* **57**, (2021).

5. Asmundson, G. J. G. & Taylor, S. Coronaphobia: Fear and the 2019-nCoV outbreak. *Journal of Anxiety Disorders* **70**, 102196 (2020).

6. Hwang, T.-J., Rabheru, K., Peisah, C., Reichman, W. & Ikeda, M. Loneliness and social isolation during the COVID-19 pandemic. *Int Psychogeriatr* 1–4 doi:10.1017/S1041610220000988.

7. Brandt, L., Liu, S., Heim, C. & Heinz, A. The effects of social isolation stress and discrimination on mental health. *Transl Psychiatry* **12**, 398 (2022).

8. McDaniels, B. & Subramanian, I. Social isolation, loneliness and mental health sequelae of the Covid-19 pandemic in Parkinson's disease. *Int Rev Neurobiol* (2022) doi:10.1016/bs.irn.2022.03.003.

9. Serafini, G. *et al.* The psychological impact of COVID-19 on the mental health in the general population. *QJM* **113**, 531–537 (2020).

10. Why does COVID-19 disproportionately affect older people? - PMC. https://www.ncbi.nlm.nih.gov/pmc/articles/PMC7288963/.

11. Lowe, C. *et al.* Impact of the COVID-19 Pandemic on Canadian Social Connections: A Thematic Analysis. *J Soc Pers Relat* 02654075221113365 (2022) doi:10.1177/02654075221113365.

For peer review only

12. Impact of the COVID-19 Pandemic on Loneliness and Social Isolation: A Multi-Country Study - PMC. https://www.ncbi.nlm.nih.gov/pmc/articles/PMC8508181/.

13. Sayin Kasar, K. & Karaman, E. Life in lockdown: Social isolation, loneliness and quality of life in the elderly during the COVID-19 pandemic: A scoping review. *Geriatr Nurs* **42**, 1222–1229 (2021).

14. Harandi, T. F., Taghinasab, M. M. & Nayeri, T. D. The correlation of social support with mental health: A meta-analysis. *Electron Physician* **9**, 5212–5222 (2017).

15. Li, F. *et al.* Effects of sources of social support and resilience on the mental health of different age groups during the COVID-19 pandemic. *BMC Psychiatry* **21**, 16 (2021).

16. Roohafza, H. R. *et al.* What's the role of perceived social support and coping styles in depression and anxiety? *J Res Med Sci* **19**, 944–949 (2014).

17. Scardera, S. *et al.* Association of Social Support During Adolescence With Depression, Anxiety, and Suicidal Ideation in Young Adults. *JAMA Netw Open* **3**, e2027491 (2020).

18. Börsch-Supan, A. Survey of Health, Ageing and Retirement in Europe (SHARE) Wave 9. COVID-19 Survey 2. (2022) doi:10.6103/SHARE.W9CA.800.

19. Börsch-Supan, A. *et al.* Data Resource Profile: the Survey of Health, Ageing and Retirement in Europe (SHARE). *Int J Epidemiol* **42**, 992–1001 (2013).

20. Kim, H.-Y. Statistical notes for clinical researchers: Risk difference, risk ratio, and odds ratio. *Restor Dent Endod* **42**, 72–76 (2017).


For peer review only

21. Riecher-Rössler, A. Sex and gender differences in mental disorders. *The Lancet Psychiatry* **4**, 8–9 (2017).

22. McLean, C. P., Asnaani, A., Litz, B. T. & Hofmann, S. G. Gender Differences in Anxiety Disorders: Prevalence, Course of Illness, Comorbidity and Burden of Illness. *J Psychiatr Res* **45**, 1027–1035 (2011).

23. McNab, S. *et al.* Comment: silent burden no more: a global call to action to prioritize perinatal mental health. *BMC Pregnancy and Childbirth* **22**, 308 (2022).

24. Grey, I. *et al.* The role of perceived social support on depression and sleep during the COVID-19 pandemic. *Psychiatry Res* **293**, 113452 (2020).

25. Guo, K. *et al.* Assessing social support impact on depression, anxiety, and stress among undergraduate students in Shaanxi province during the COVID-19 pandemic of China. *PLoS One* **16**, e0253891 (2021).

26. Ortiz-Calvo, E. *et al.* The role of social support and resilience in the mental health impact of the COVID-19 pandemic among healthcare workers in Spain. *J Psychiatr Res* **148**, 181–187 (2022).

27. Długosz, P., Liszka, D. & Yuzva, L. The Link between Subjective Religiosity, Social Support, and Mental Health among Young Students in Eastern Europe during the COVID-19 Pandemic: A Cross-Sectional Study of Poland and Ukraine. *Int J Environ Res Public Health* **19**, 6446 (2022).

28. Miani, C., Wandschneider, L., Niemann, J., Batram-Zantvoort, S. & Razum, O. Measurement of gender as a social determinant of health in epidemiology—A scoping review. *PLoS One* **16**, e0259223 (2021).

29. Bolte, G. *et al.* Integrating Sex/Gender into Environmental Health Research: Development of a Conceptual Framework. *Int J Environ Res Public Health* **18**, 12118 (2021).

For peer review only

30. Short, S. E., Yang, Y. C. & Jenkins, T. M. Sex, Gender, Genetics, and Health. *Am J Public Health* **103**, S93–S101 (2013).

For peer review only

Figure legends:

**Figure 1: Percentage of participants who reported having depression, Nervousness, Loneliness, and Sleep Trouble by country.**

As shown in figure 1, the percentage of self-reported depression was over 40% in Portugal (46.44%), Bulgaria (42.5%), and Hungary (42.33%). Portugal (53.37%), Bulgaria (50.83%) also had the highest percentages of self-reported nervousness. The percentage of loneliness was highest in Greece (52.91%) and that of sleep trouble was highest in Estonia (49.13%).

For peer review only

**Figure 2: comparison of the total number of conditions reported by male and female participants.**

Figure 2 shows that about a quarter of the men and women reported at least one of the four conditions. However, the percentage of reporting more than one condition was higher among women than men. For example, 10.72% of the women reported having all four conditions compared with 4.81% among men.

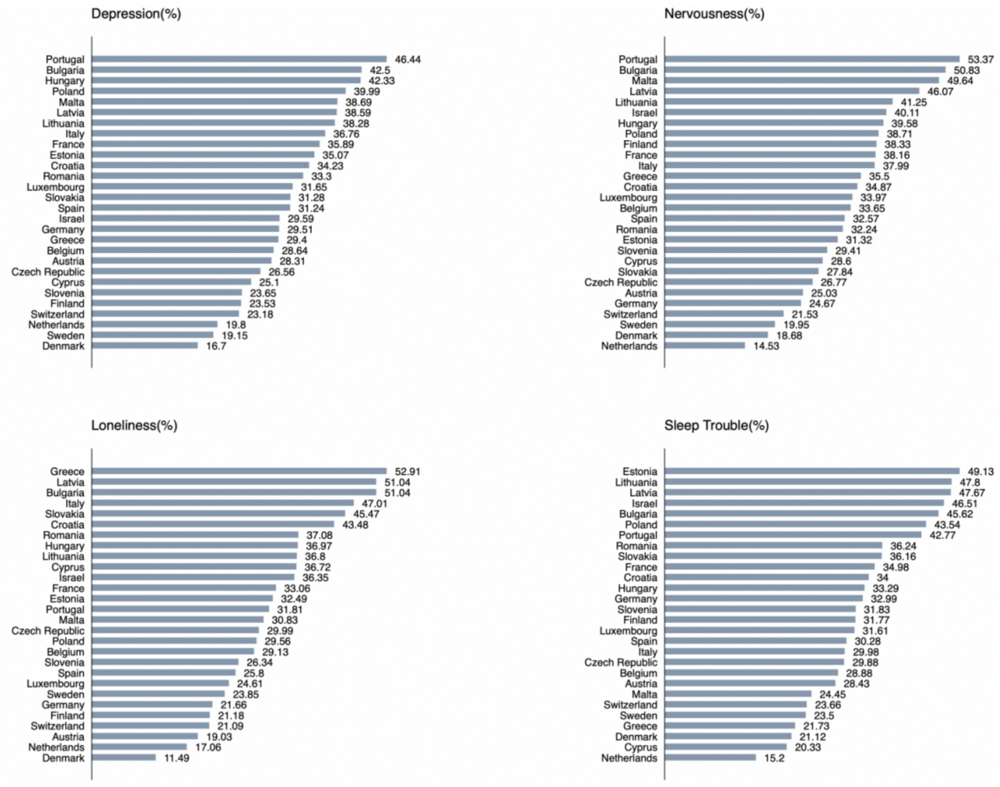


Percentage of participants who reported having depression, Nervousness, Loneliness, and Sleep Trouble by country.

165x129mm (220 x 220 DPI)

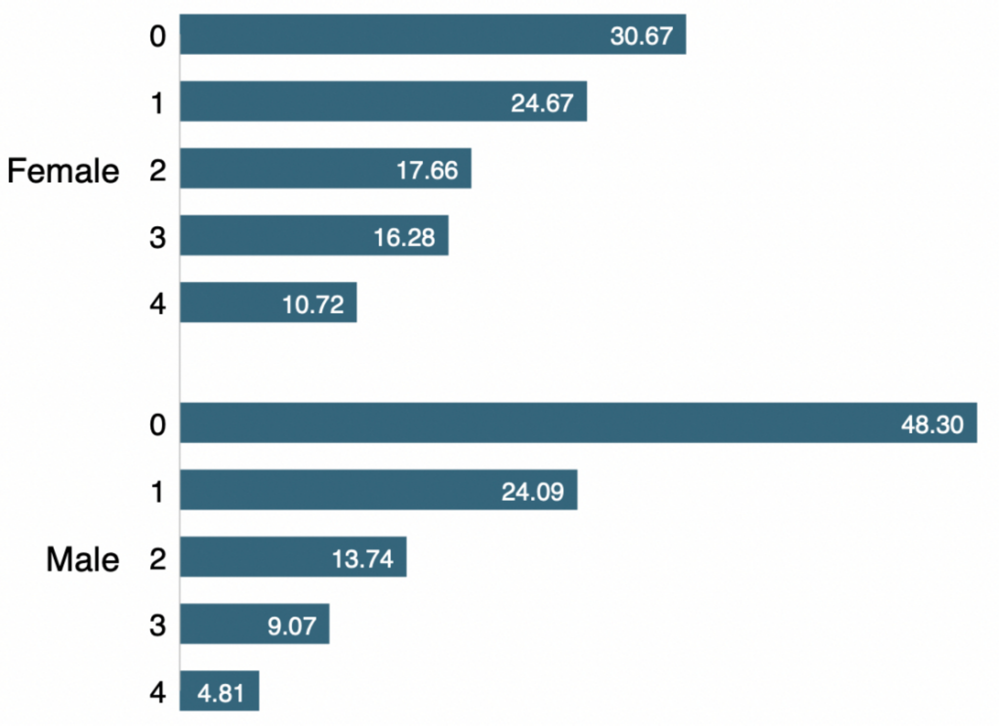


comparison of the total number of conditions reported by male and female participants.

118x86mm (220 x 220 DPI)

Appendix

Table 1a: Sample description (Share 8).

| age | Freq. | Percent | Cum. |
|---|---|---|---|
| 65-69 years | 9857 | 26.92 | 26.92 |
| 70-74 years | 9719 | 26.54 | 53.46 |
| 75-79 years | 7508 | 20.50 | 73.96 |
| 80-84 years | 5399 | 14.74 | 88.70 |
| 85+ | 4138 | 11.30 | 100.00 |
| | | | |
| Sex | Freq. | Percent | Cum. |
| Male | 15719 | 42.92 | 42.92 |
| Female | 20902 | 57.08 | 100.00 |
| | | | |
| Employment status | Freq. | Percent | Cum. |
| Retired | 32195 | 88.03 | 88.03 |
| Employment | 1626 | 4.45 | 92.48 |
| Unemployed | 79 | 0.22 | 92.69 |
| Permanently sick or disabled | 298 | 0.81 | 93.51 |
| Homemaker | 1980 | 5.41 | 98.92 |
| Other | 395 | 1.08 | 100.00 |
| | | | |
| Rating of subjective health | Freq. | Percent | Cum. |
| Excellent | 1266 | 3.46 | 3.46 |
| Very good | 4789 | 13.09 | 16.55 |
| Good | 14290 | 39.05 | 55.60 |
| Fair | 12106 | 33.08 | 88.69 |
| Poor | 4140 | 11.31 | 100.00 |
| | | | |
| Country | | | |
| Austria | 1943 | 5.31 | 5.31 |
| Germany | 1466 | 4.00 | 9.31 |
| Sweden | 856 | 2.34 | 11.65 |
| Netherlands | 592 | 1.62 | 13.26 |
| Spain | 1545 | 4.22 | 17.48 |
| Italy | 2528 | 6.90 | 24.38 |
| France | 1458 | 3.98 | 28.37 |

For peer review only

| | | | |
|---|---|---|---|
| Denmark | 1143 | 3.12 | 31.49 |
| Greece | 2515 | 6.87 | 38.36 |
| Switzerland | 1452 | 3.96 | 42.32 |
| Belgium | 2427 | 6.63 | 48.95 |
| Israel | 1132 | 3.09 | 52.04 |
| Czech Republic | 1808 | 4.94 | 56.98 |
| Poland | 1795 | 4.90 | 61.88 |
| Luxembourg | 581 | 1.59 | 63.46 |
| Hungary | 715 | 1.95 | 65.42 |
| Portugal | 860 | 2.35 | 67.76 |
| Slovenia | 2313 | 6.32 | 74.08 |
| Estonia | 3012 | 8.22 | 82.31 |
| Croatia | 1305 | 3.56 | 85.87 |
| Lithuania | 774 | 2.11 | 87.98 |
| Bulgaria | 480 | 1.31 | 89.29 |
| Cyprus | 490 | 1.34 | 90.63 |
| Finland | 855 | 2.33 | 92.97 |
| Latvia | 623 | 1.70 | 94.67 |
| Malta | 548 | 1.50 | 96.16 |
| Romania | 919 | 2.51 | 98.67 |
| Slovakia | 486 | 1.33 | 100.00 |

For peer review only

BMJ Open

# Social support and psychosocial well-being among older adults in Europe during the COVID-19 pandemic: a cross-sectional study

| | |
|---|---|
| Journal: | *BMJ Open* |
| Manuscript ID | bmjopen-2022-071533.R2 |
| Article Type: | Original research |
| Date Submitted by the Author: | 09-Jun-2023 |
| Complete List of Authors: | Lu, Ji; Tongji Medical College,<br>Xiong, Juyang; Huazhong University of Science and Technology Tongji Medical College, School of Medicine and Health Management<br>Tang, Shangfeng; Tongji Medical College of Huazhong University of Science and Technology, Health mangament<br>Bishwajit, Ghose; University of Ottawa, Faculty of Health Sciences;<br>Guo, Shuyan; National Institute of Hospital Administration |
| <b>Primary Subject Heading</b>: | Mental health |
| Secondary Subject Heading: | Epidemiology |
| Keywords: | COVID-19, MENTAL HEALTH, SOCIAL MEDICINE |
| | |

SCHOLARONE™
Manuscripts

*I, the Submitting Author has the right to grant and does grant on behalf of all authors of the Work (as defined in the below author licence), an exclusive licence and/or a non-exclusive licence for contributions from authors who are: i) UK Crown employees; ii) where BMJ has agreed a CC-BY licence shall apply, and/or iii) in accordance with the terms applicable for US Federal Government officers or employees acting as part of their official duties; on a worldwide, perpetual, irrevocable, royalty-free basis to BMJ Publishing Group Ltd ("BMJ") its licensees and where the relevant Journal is co-owned by BMJ to the co-owners of the Journal, to publish the Work in this journal and any other BMJ products and to exploit all rights, as set out in our licence.*

*The Submitting Author accepts and understands that any supply made under these terms is made by BMJ to the Submitting Author unless you are acting as an employee on behalf of your employer or a postgraduate student of an affiliated institution which is paying any applicable article publishing charge ("APC") for Open Access articles. Where the Submitting Author wishes to make the Work available on an Open Access basis (and intends to pay the relevant APC), the terms of reuse of such Open Access shall be governed by a Creative Commons licence – details of these licences and which Creative Commons licence will apply to this Work are set out in our licence referred to above.*

*Other than as permitted in any relevant BMJ Author's Self Archiving Policies, I confirm this Work has not been accepted for publication elsewhere, is not being considered for publication elsewhere and does not duplicate material already published. I confirm all authors consent to publication of this Work and authorise the granting of this licence.*

For peer review only

**Social support and psychosocial well-being among older adults in Europe during the COVID-19 pandemic: a cross-sectional study**

**Lu Ji[1*], PhD; Juyang Xiong[1*], PhD; Shangfeng Tang[1], PhD; Ghose Bishwajit[2], PhD; Shuyan Guo[3]**

1 Lu Ji， School of Medicine and Health Management, Tongji Medical College of Huazhong University of Science and Technology, Wuhan, China. Jilu2020@hust.edu.cn

1 Juyang Xiong, School of Medicine and Health Management, Tongji Medical College of Huazhong University of Science and Technology, Wuhan, China. xiongjuyang@hust.edu.cn

1 Shangfeng Tang, School of Medicine and Health Management, Tongji Medical College of Huazhong University of Science and Technology, Wuhan, China. Sftang2018@hust.edu.cn

2 Ghose Bishwajit, Faculty of Health Sciences, University of Ottawa, Ottawa, Ontario, Canada. Brammaputram@gmail.com

3 Shuyan Guo, National Institute of Hospital Administration, National Health Commission, Beijing, China, guoshuyan@niha.org.cn

*Lu Ji and Juyang Xiong contributed equally

Correspondence to Shuyan Guo, guoshuyan@niha.org.cn

Word count: 3800.

For peer review only

**Abstract**: The objective of the study was to identify the association between social support and psychosocial well-being among men and women aged over 65 years in Europe during the COVID-19 pandemic.

**Methods**: Cross-sectional data on 36,621 men (n=15,719) and women (n=20,902) aged 65 years or higher were obtained from the ninth round of the Survey of Health, Ageing and Retirement in Europe (SHARE). The outcomes were measured by psychosocial well-being reflected with self-reported depression, nervousness, loneliness and sleep disturbances. Social support was measured in terms of receiving help from own children, relatives, and neighbours/friends/colleagues since the pandemic outbreak.

**Result**: About one-third of the participants reported depression (31.03%), nervousness (32.85%), loneliness (32.23%), and sleep trouble (33.01%). The results of multivariable regression analysis revealed that social support was a protective factor to psychological well-being. For instance, receiving help from own children [RD= -0.13, 95% CI = -0.14, -0.12], relatives [RD= -0.08, 95% CI = -0.11, -0.06], neighbours/friends/colleagues [RD= -0.11, 95% CI = -0.13, -0.09], and receiving home care [RD= -0.20, 95% CI = -0.22, -0.18] showed significantly lower risk difference for depression. Similar findings were noted for loneliness, nervousness, and sleep trouble as well, with the risk difference being slightly different for men and women in the gender-stratified analysis. For instance, the risk difference in depression for receiving help from own children was -0.10 [95% confidence interval = -0.12, -0.08] among men compared with -0.12 [95% confidence interval = -0.14, -0.11] among women. The risk differences in the outcome measures were calculated using Generalized Linear Model for binomial family.

For peer review only - http://bmjopen.bmj.com/site/about/guidelines.xhtml

**Conclusion**: Findings of the present study highlight a protective role of social support on psychological well-being among both men and women. Developing strategies to promote social support, especially among older adults, may mitigate the rising burden of psychological illness during the COVID-19 pandemic.

**Keywords**: COVID-19; aging health; Mental health; Social support; Well-being.

For peer review only

**Strengths and limitations**

•This study focused on the association between social support and psychosocial well-being among older adults during the COVID-19 pandemic in EU countries.

•The analysis conducted using multiple dimensions of psychosocial well-being and social support.

• The cross-sectional nature of the surveys prevents establishing causal relationships between outcome and explanatory variables.

• Inclusion of all relevant sociocultural factors was not possible due to the secondary analysis.

## Introduction

The COVID-19 pandemic has become one of the most devastating health and social crises in generations due to its long-term impacts on physical and mental health, social life and livelihoods

worldwide.[1–3] A pervasive crisis is the emergence of a phenomenon known as "coronaphobia" - fear or anxiety related to the virus and its potential impacts on the psychosocial well-being of individuals and communities at large.[4,5] A growing number of studies have highlighted the prevalence of mental illness in the context of lifestyle such as higher consumption of psychoactive drugs and widespread social isolation and loneliness.[6–9] The fear of contracting the virus has compelled people to stay at home for an extended period of time, particularly affecting older adults who face an elevated risk of severe illness from the virus.[10]

For peer review only

The measures to control the COVID-19 pandemic, such as physical distancing, have caused disruptions to a healthy and active lifestyle, as well as the opportunities to maintain social connections and seek help in times of need.[11] The impact of social isolation and loneliness on mental health has been an increasing concern in the wake of the global pandemic, as socialization opportunities have become limited and physical distancing rules have been put in place.[12] As such, there has been a dramatic decrease in social interaction and support, which is particularly concerning for peoples' psychosocial well-being during the global health crisis. Prolonged isolation can lead to feelings of loneliness, depression and anxiety and can take a toll on mental health of older adults due to their higher dependence on physical and emotional care.[13,14]

The feeling of depression and loneliness are common mental health concerns that can affect people from all walks of life, especially among older adults. This is due to a range of factors, including changes in physical health and lifestyle, increased isolation and loneliness, financial concerns, and difficulty in adapting to the technological advances of modern life. The COVID-19 pandemic has fuelled social isolation and loneliness mainly through fewer opportunities for meeting friends and families, organizing or participating in social gatherings and reduced social support.[15] Social support, which is a key aspect of social capital, has been shown to be strongly correlated with mental health in previous studies.[16,17] Perceived social support refers to the

For peer review only

subjective availability of psychological, physical, and financial help from social relationships such as family, friends, neighbours, and community members that is available in times of need. Social support plays an important role in mitigating the impact of social isolation and can help reduce stress, improve mood, and promote positive coping mechanisms.[18] Social support also provides a sense of belonging and connection, which can offset feelings of loneliness and isolation. Several previous studies have demonstrated the link between social relationships and support and mental health.[16,18,19] However, there is not enough evidence regarding this relationship among older adults in European countries in the context of the COVID-19 pandemic. The present study therefore investigates the relationship between social support and psychosocial well-being among older adults in Europe using the data from the Survey of Health Ageing, and Retirement (WAVE 9).

## Methods

### Data source

Data for this study were derived from the ninth wave of the Survey of Health Ageing, and Retirement in Europe (SHARE).[20] The survey aims to provide a framework for researchers to better understand population ageing and covers most of the European Union and Israel. SHARE has been adopted as a model by a number of ageing surveys throughout the world and is harmonized with the US Health and Retirement Study (HRS) and the English Longitudinal Study of Ageing (ELSA).[21] The SHARE survey collects data on a diverse range of health and socioeconomic indicators among individuals including demographics, living conditions, income and employment status, mental health, satisfaction with health conditions and social networks. The

For peer review only

ninth wave of the Survey included questions relevant to COVID-19, such as receiving support from social connections to obtain basic necessities. Data for this survey were collected via computer-assisted telephone interviews between June and August of 2021 among individuals aged 50 or older. For the purpose of the present study, the analysis was limited to participants aged 65 years or older only.

**Description of the variables**

The outcome measures included four indicators of psychosocial well-being assessed by self-reported nervousness, depression, sleep disturbances and loneliness. These indicators were assessed by the following questions: 1) felt nervous in last month (Yes/No), 2) sad or depressed in last month (Yes/No), 3) trouble sleeping recently (Yes/No), 4) how often feelings of loneliness (Often/Some of the times/Hardly ever or never). For loneliness, the answers were recoded to 'Yes' for responses 'Often/Some of the times' and to 'No' for responses 'Hardly ever or never'. Social support was assessed in terms of receiving help in obtaining necessities during the pandemic from own children, relatives, and neighbours/friends/colleagues to obtain necessities since the outbreak. These indicators were assessed by the following questions: 1) help received from own children to obtain necessities since the outbreak (Yes/No), 2) help received from other relatives to obtain necessities since the outbreak (Yes/No), 3) help received from neighbours/friends/colleagues to obtain necessities (Yes/No), 4) received regular home care during last 3 months (Yes/No). Apart from the indicators of social support, the analysis also included several socioeconomic variables based on their known association with mental health status such as age (65-69 years/ 70-74 years/

For peer review only

75-79 years/ 80-84 years/ 85+ years), sex (male/female), employment situation (Retired/ Employed or self-employed/ Unemployed/ Permanently sick or disabled? Homemaker/ Other), and country of residence (list of countries provided in appendix).

## Statistical Analysis

All statistical analyses were conducted using Stata 17 (Stata Corp., College Station, Texas). The dataset was first examined to make sure the sample population was defined correctly (65 years and older) and there were no missing values and outliers. Descriptive statistics were carried out to calculate the percentage of participants reporting nervousness, depression, loneliness, and sleep trouble. Sex and country-level percentages were presented as bar charts. In the next step, we conducted generalized linear models (GLMs) for binomial family to measure the risk difference (with 95% confidence intervals) in the four outcome variables (nervousness, depression, loneliness, and sleep trouble). The results of GLMs were presented as risk difference (RD), defined as the risk of a condition between an exposed group and an unexposed group.[22] A positive RD value indicates a higher risk by the exposure, whereas a negative one indicates a lower risk. Given the well-documented gender differences in mental health outcomes, the analysis was stratified by male and female participants for each outcome variable. The level of statistical significance for all associations was set at 0.05.

**Patient and public involvement**: No patient involved.

For peer review only

## Results

The sociodemographic characteristics of the sample population are available in the appendix file. A greater percentage of the participants were in the age bracket of 65-69 years (n= 26.92%), female (n= 57.08%), retired (n= 88.03%), and rated general health status as good (n= 39.05%). Sample size for the participating countries was as follows: Austria (n= 1943), Germany (n= 1466), Sweden (n= 856), Netherlands (n= 592), Spain (n= 1545), Italy (n= 2528), France (n= 1458), Denmark (n= 1143), Greece (n= 2515), Switzerland (n= 1452), Belgium (n= 2427), Israel (n= 1132), Czech Republic (n= 1808), Poland (n= 1795), Luxembourg (n= 581), Hungary (n= 715), Portugal (n= 860), Slovenia (n= 2313), Estonia (n= 3012), Croatia (n= 1305), Lithuania (n= 774), Bulgaria (n= 480), Cyprus (n= 490), Finland (n= 855), Latvia (n= 623), Malta (n= 548), Romania (n= 919), Slovakia (n= 486).

Among the four psychosocial well-being indicators, sleep trouble (33.01%) was the most commonly reported one (table 1), followed by nervousness (32.85%) and loneliness (32.23%). As described in table 1, the percentage of reporting depression (77.08% vs 62.88%), nervousness (73.56% vs 62.33%), and loneliness (76.43% vs 61.27%) was higher in men, while that of sleep trouble was higher in women (25.73% vs 38.49%).

For peer review only

**Table 1: Percentage of participants who reported having depression, Nervousness, Loneliness, and Sleep Trouble by sex.**

| | Percentage of Total | Percentage of total Men | Percentage of total Women |
|---|---|---|---|
| Depression | 31.03 | 22.92 | 37.12 |
| Nervousness | 32.85 | 26.44 | 37.67 |
| Loneliness | 32.23 | 23.57 | 38.73 |
| Sleep trouble | 33.01 | 25.73 | 38.49 |

**Figure 1: Percentage of participants who reported having depression, Nervousness, Loneliness, and Sleep Trouble by country.**

For peer review only

**Figure 2: comparison of the total number of conditions reported by male and female participants.**

The results of multivariable regression analysis on the association between self-reported depression, nervousness, loneliness, sleep trouble and social support are shown in table 2. Receiving help from own children [RD= -0.13, 95% confidence interval = -0.14, -0.12], relatives [RD= -0.08, 95% confidence interval = -0.11, -0.06], neighbours/friends/colleagues [RD= -0.11, 95% confidence interval = -0.13, -0.09], and receiving home care [RD= -0.20, 95% confidence interval = -0.22, -0.18] showed significantly lower risk difference for depression, with the differences being slightly higher among women compared with men. For instance, the risk difference in depression for receiving help from own children was -0.10 [95% confidence interval = -0.12, -0.08] among men compared with -0.12 [95% confidence interval = -0.14, -0.11] among women.

Receiving help from own children [RD= -0.10, 95% confidence interval = -0.12, -0.09], relatives [RD= -0.05, 95% confidence interval = -0.07, -0.03], neighbours/friends/colleagues

For peer review only

[RD= -0.08, 95% confidence interval = -0.10, -0.06], and receiving home care [RD= -0.15, 95% confidence interval = -0.17, -0.13] showed significantly lower risk difference for nervousness. In the gender-stratified analysis, receiving help from own children showed a relatively higher risk difference among women, whereas receiving help from neighbours/friends/colleagues showed a relatively higher risk difference among men.

Receiving help from own children [RD= -0.13, 95% confidence interval = -0.14, -0.11] relatives [RD= -0.08, 95% confidence interval = -0.10,-0.05] neighbours/friends/colleagues[RD= -0.12, 95% confidence interval = -0.13, -0.10], and receiving home care [RD= -0.12, 95% confidence interval = -0.14,-0.10] showed significantly lower risk difference for loneliness. The risk difference was relatively higher among men who reported receiving help from own children, relatives, and neighbours/friends/colleagues.

Receiving help from own children [RD= -0.10, 95% confidence interval = -0.11,-0.09], relatives [RD= -0.05, 95% confidence interval = -0.08, -0.03], neighbours/friends/colleagues [-0.07, 95% confidence interval = -0.09,-0.05], and receiving home care [RD= -0.12, 95% confidence interval =-0.14,-0.10] showed significantly lower risk difference for sleep trouble. In the gender-stratified analysis, receiving help from own children and from neighbours/friends/colleagues showed a relatively higher risk difference among men.

For peer review only

Table 2: Association between social support and self-reported depression, nervousness, loneliness and sleep trouble.

| | Full sample | Men | Women |
|---|---|---|---|
| | | | |
| **Depression** | | | |
| **Received help from own children (Yes)** | ref | ref | ref |
| No | -0.13*** [-0.14,-0.12] | -0.10*** [-0.12,-0.08] | -0.12*** [-0.14,-0.11] |
| **Received help from relatives (Yes)** | ref | ref | ref |
| No | -0.08*** [-0.11,-0.06] | -0.07*** [-0.11,-0.03] | -0.08*** [-0.11,-0.05] |
| **Received help from neighbors/friends/colleagues (yes)** | ref | ref | ref |
| No | -0.11*** [-0.13,-0.09] | -0.09*** [-0.12,-0.06] | -0.11*** [-0.13,-0.08] |
| **Received home care (Yes)** | ref | ref | ref |
| No | -0.20*** [-0.22,-0.18] | -0.19*** [-0.22,-0.15] | -0.19*** [-0.21,-0.16] |
| | | | |
| **Nervousness** | | | |
| **Received help from own children (Yes)** | ref | ref | ref |

For peer review only

| | | | |
|---|---|---|---|
| No | -0.10*** [-0.12,-0.09] | -0.10*** [-0.11,-0.08] | -0.09*** [-0.11,-0.08] |
| **Received help from relatives (Yes)** | ref | ref | ref |
| No | -0.05*** [-0.07,-0.03] | -0.04* [-0.08,-0.01] | -0.05*** [-0.08,-0.02] |
| **Received help from neighbors/friends/colleagues (yes)** | ref | ref | ref |
| No | -0.08*** [-0.10,-0.06] | -0.06*** [-0.09,-0.02] | -0.09*** [-0.11,-0.06] |
| **Received home care (Yes)** | ref | ref | ref |
| No | -0.15*** [-0.17,-0.13] | -0.15*** [-0.18,-0.11] | -0.15*** [-0.17,-0.12] |
| | | | |
| **Loneliness** | | | |
| **Received help from own children (Yes)** | ref | ref | ref |
| No | -0.13*** [-0.14,-0.11] | -0.09*** [-0.11,-0.08] | -0.12*** [-0.13,-0.10] |
| **Received help from relatives (Yes)** | ref | ref | ref |
| No | -0.08*** [-0.10,-0.05] | -0.07*** [-0.11,-0.03] | -0.08*** [-0.10,-0.05] |
| | | | |
| **Received help from neighbors/friends/colleagues (yes)** | ref | ref | ref |
| No | -0.12*** [-0.13,-0.10] | -0.07*** [-0.10,-0.04] | -0.12*** [-0.15,-0.10] |

For peer review only

| | | | |
|---|---|---|---|
| **Received home care (Yes)** | ref | ref | ref |
| No | -0.12*** [-0.14,-0.10] | -0.13*** [-0.16,-0.10] | -0.10*** [-0.12,-0.07] |
| | | | |
| **Sleep trouble** | | | |
| **Received help from own children (Yes)** | ref | ref | ref |
| No | -0.10*** [-0.11,-0.09] | -0.07*** [-0.09,-0.05] | -0.09*** [-0.10,-0.08] |
| | | | |
| **Received help from relatives (Yes)** | ref | ref | ref |
| No | -0.05*** [-0.08,-0.03] | -0.06** [-0.10,-0.02] | -0.04** [-0.07,-0.01] |
| **Received help from neighbors/friends/colleagues (yes)** | ref | ref | ref |
| No | -0.07*** [-0.09,-0.05] | -0.06*** [-0.09,-0.03] | -0.07*** [-0.09,-0.04] |
| **Received home care (Yes)** | ref | ref | ref |
| No | -0.12*** [-0.14,-0.10] | -0.13*** [-0.16,-0.10] | -0.10*** [-0.12,-0.07] |

N.B. cells represent risk differences with 95% confidence intervals. All models are adjusted for age, sex, employment status, subjective health status, and country of residence. Level of significance: * $p < 0.05$, ** $p < 0.01$, *** $p < 1$

For peer review only

## Discussion

Given the rising burden of mental illness during the COVID-19 pandemic, it's important to explore the potential role of the social determinants that can affect mental health, especially among older adults who share a greater vulnerability to both COVID-19 and mental health conditions. The present study aimed to assess the relationship between social support and indicators of psychosocial well-being among older men and women in 27 countries in Europe and Israel using data from the Survey of Health Ageing, and Retirement. Our findings suggest that about one-third of the participants reported depression, nervousness, loneliness, and sleep trouble, with the percentage of all four conditions being relatively higher among women. Overall, about a quarter of the participants reported at least one, and about 8% reported having all of the four conditions. However, the percentage of reporting multiple conditions was higher among women than among men, indicating a greater burden of psychological issues among women. Previous studies have reported a higher burden of mental health issues among women and attempted to explain the gender differences in mental disorders through various biological (pregnancy, hormonal) and psychosocial factors (self-esteem, social empowerment).[23–25] Apart from the gender differences, we observed remarkable inter-country differences in the corresponding percentages as well. For instance, the percentage of depression ranged from 46.44% in Portugal to 16.7% in Denmark, nervousness from 53.37% in Portugal to 14.53% in the Netherlands, loneliness from 52.91% in Greece to 11.49% in Denmark, and sleep trouble from 49.13% in Estonia to 15.2% in the Netherlands. The country-level disparities might be rooted in the health policies related to the

For peer review only

pandemic and health and social conditions that are relevant to mental health in individual countries that should be investigated through more in-depth studies.

Regarding the association between social support in obtaining the necessities during the pandemic and the outcome measures, receiving help from own children, relatives, neighbours/friends/colleagues, and home care showed significantly lower risks of depression, nervousness, loneliness, and sleep trouble compared with those didn't receive any help. Several previous studies have reported the positive association between social support and psychosocial well-being through a social capital lens. According to a cohort study conducted in Quebec, Canada, adolescents who felt they had more social support one year later reported having fewer mental health issues, concluding that perceived social support, especially in those who have mental health issues in youth, may shield against mental health issues as people enter adulthood.[19] A meta-analysis involving 64 studies reported a mean effect size of 0.356, indicating a moderate effect size of social support on mental health.[16] Another study conducted in Lebanon using structured questionnaires for depression (Patient Health Questionnaire), loneliness (UCLA Loneliness Scale) and social support (Multidimensional Scale of Perceived Social Support) found that the rates of depression and loneliness were significantly higher among participants who experienced self-isolation compared to those who didn't.[26] The same study reported that the risk of depressive symptoms and poor sleep quality were respectively 63% and 52% lower among individuals who reported higher levels of social support compared to those with low perceived social support.[26] In

For peer review only

the context of the COVID-19 pandemic, receiving social support was reported to be inversely associated with mental health problems in several countries including China,[27] Spain,[28] Poland and Ukraine.[29]

The findings of the current study are consistent with previous ones regarding the positive role of social support on psychological well-being. Social support can take many forms, from family and friends to community groups and online forums, and serves the purpose of providing a sense of connection and belonging and improving coping skills, which can be vital for people who are feeling isolated. Lack of social support can therefore exacerbate the effects of social isolation and lead to further mental health problems. Arguably, the social stressors associated with the pandemic can lead to short- and long-term psychological issues such as anxiety and depression, which in turn can contribute to feelings of loneliness and isolation. With the increasing demands of modern life, individuals are becoming more aware of the protective effects of relationships on health and overall quality of life as it can act as a buffer against social stressors and promote positive mental well-being. Mental health experts have also alarmed about the dangers of social isolation since the start of the COVID-19 pandemic. Policymakers and social researchers should therefore aim to develop innovative strategies for promoting this invaluable resource to build more resilient communities that can help mitigate the long-term mental health impacts of the pandemic.

For peer review only

As far as we are concerned, this is the first study to focus on the association between social support and psychosocial well-being among the older adults during the COVID-19 pandemic in EU countries. The sample size was large and the data were obtained from a reputed population survey- the largest pan-European study that provides internationally comparable data on a large number of health and social indicators. The present study included participants aged 65 years and older, and the analysis was conducted using several dimensions of psychosocial well-being and social support. An important aspect of the study was the gender-stratified analysis of the association between psychosocial well-being and social support, which allowed a better understanding of the differences in the way men and women may respond to certain types of social support. Previous studies have underscored that gender-stratified analysis allows better insights into how biological, physiological, behavioural, environmental, and social factors interact differently in people of different genders.[30–32] As such, gender-stratified analysis is becoming increasingly important as researchers strive to improve the understanding of the unique healthcare needs of men and women. The importance of social support for psychological well-being is a well-established finding in previous literature. However, in the present study, it was not possible to directly compare this phenomenon with the pre-pandemic time due to the unavailability of similar data. This study has several important limitations to consider as well. Firstly, the data used in this analysis are cross-sectional and therefore no causal relationship can be established between the outcome and explanatory variables. Since the analysis was based on pre-existing data, the authors had no control over the selection and measurement of the study variables. As a result, not all dimensions of social support that are relevant to psychosocial well-being were possible to include

For peer review only

in the analysis. Secondly, there was no specific information on what type of support was received by the participants from their contacts (e.g. financial, activities of daily living, emotional, healthcare visits) which could have produced a more detailed picture of the association between social support and psychosocial well-being. The analysis cannot clarify the nature or quality of the relationships as well. Someone receiving all the necessary support during the pandemic may experience higher health benefits compared to those receiving the support occasionally. The measures of psychosocial well-being are also self-reported and therefore remain subject to reporting bias. Future studies should focus on addressing these limitations and use more objective indicators of social support and mental health. Regardless of these limitations, these findings make an important contribution to the literature on the positive role of social capital on psychosocial well-being. As the pandemic is starting to exert long-term social crises, which in turn are affecting people's health status, policymakers should attempt to make socially tailored decisions to minimize the effect of social isolation and provide support, especially for the most vulnerable communities.

**Conclusion**

According to the findings, about one-third of the participants reported having nervousness, depression, loneliness, and sleep trouble. A quarter of them reported at least one, and about 8% reported having all of the four conditions, with the percentages being relatively higher among women than among men. A considerable inter-country difference was found in the corresponding percentages as well. Receiving help from own children, relatives, neighbours/friends/colleagues, and home care showed significantly lower risks of depression, nervousness, loneliness, and sleep

For peer review only

trouble compared with those who didn't receive any help. Interestingly, the risk differences for all four indicators varied between men and women concerning the sources of social support such that the benefits are generally more pronounced among men than women.

**Author Contributions**: Conceptualization, L.J.; methodology, J.X., G.B., S.T., S.G.; software, J.X., G.B., S.T., S.G.; formal analysis, J.X., G.B., S.T., S.G.; data curation, G.B.; writing—original draft preparation, L.J., J.X., G.B., S.T., S.G.; writing—review and editing L.J., J.X., G.B., S.T., S.G.; All authors have read and agreed to the published version of the manuscript.

**Funding**: This work was funded by grant 2022YFE0133000 from the National Key R&D Program of China, grant 72004073 from the National Natural Science Foundation of China, and grant 20YJC630134 from the Chinese Ministry of Education of Humanities and Social Science project. The funders had no role in the design or decision to publish the manuscript.

**Conflict of interest**: None to declare.

**Data availability**: Survey of Health, Ageing and Retirement in Europe. DOI: 10.6103/SHARE.w9ca.800. 2022-02-10

**Ethics Approval/Patient consent for publication**: Not applicable, data are secondary.

For peer review only

**References**

1. Dubey, S. *et al.* Psychosocial impact of COVID-19. *Diabetes Metab Syndr* **14**, 779–788 (2020).

2. Etowa, J. *et al.* Social determinants of the disproportionately higher rates of COVID-19 infection among African Caribbean and Black (ACB) population: A systematic review protocol. *J Public Health Res* **11**, 2274 (2021).

3. Hiscott, J. *et al.* The global impact of the coronavirus pandemic. *Cytokine Growth Factor Rev* **53**, 1–9 (2020).

4. Abdelghani, M., Hassan, M. S., Elgohary, H. M. & Fouad, E. Exploring the factors associated with coronaphobia among physicians during the COVID-19 outbreak in Egypt. *The Egyptian Journal of Neurology, Psychiatry and Neurosurgery* **57**, (2021).

5. Asmundson, G. J. G. & Taylor, S. Coronaphobia: Fear and the 2019-nCoV outbreak. *Journal of Anxiety Disorders* **70**, 102196 (2020).

6. Hwang, T.-J., Rabheru, K., Peisah, C., Reichman, W. & Ikeda, M. Loneliness and social isolation during the COVID-19 pandemic. *Int Psychogeriatr* 1–4 doi:10.1017/S1041610220000988.

7. Brandt, L., Liu, S., Heim, C. & Heinz, A. The effects of social isolation stress and discrimination on mental health. *Transl Psychiatry* **12**, 398 (2022).

8. McDaniels, B. & Subramanian, I. Social isolation, loneliness and mental health sequelae of the Covid-19 pandemic in Parkinson's disease. *Int Rev Neurobiol* (2022) doi:10.1016/bs.irn.2022.03.003.

9. Serafini, G. *et al.* The psychological impact of COVID-19 on the mental health in the general population. *QJM* **113**, 531–537 (2020).


For peer review only

10. Why does COVID-19 disproportionately affect older people? - PMC. https://www.ncbi.nlm.nih.gov/pmc/articles/PMC7288963/.

11. Lowe, C. *et al.* Impact of the COVID-19 Pandemic on Canadian Social Connections: A Thematic Analysis. *J Soc Pers Relat* 02654075221113365 (2022) doi:10.1177/02654075221113365.

12. Impact of the COVID-19 Pandemic on Loneliness and Social Isolation: A Multi-Country Study - PMC. https://www.ncbi.nlm.nih.gov/pmc/articles/PMC8508181/.

13. Cosco, T. D. *et al.* Ageing and Mental Health in Canada: Perspectives from Law, Policy, and Longitudinal Research. *J Popul Ageing* **15**, 863–878 (2022).

14. Rosso, A. L., Taylor, J. A., Tabb, L. P. & Michael, Y. L. MOBILITY, DISABILITY, AND SOCIAL ENGAGEMENT IN OLDER ADULTS. *J Aging Health* **25**, 617–637 (2013).

15. Sayin Kasar, K. & Karaman, E. Life in lockdown: Social isolation, loneliness and quality of life in the elderly during the COVID-19 pandemic: A scoping review. *Geriatr Nurs* **42**, 1222–1229 (2021).

16. Harandi, T. F., Taghinasab, M. M. & Nayeri, T. D. The correlation of social support with mental health: A meta-analysis. *Electron Physician* **9**, 5212–5222 (2017).

17. Li, F. *et al.* Effects of sources of social support and resilience on the mental health of different age groups during the COVID-19 pandemic. *BMC Psychiatry* **21**, 16 (2021).

18. Roohafza, H. R. *et al.* What's the role of perceived social support and coping styles in depression and anxiety? *J Res Med Sci* **19**, 944–949 (2014).

For peer review only

19. Scardera, S. *et al.* Association of Social Support During Adolescence With Depression, Anxiety, and Suicidal Ideation in Young Adults. *JAMA Netw Open* **3**, e2027491 (2020).

20. Börsch-Supan, A. Survey of Health, Ageing and Retirement in Europe (SHARE) Wave 9. COVID-19 Survey 2. (2022) doi:10.6103/SHARE.W9CA.800.

21. Börsch-Supan, A. *et al.* Data Resource Profile: the Survey of Health, Ageing and Retirement in Europe (SHARE). *Int J Epidemiol* **42**, 992–1001 (2013).

22. Kim, H.-Y. Statistical notes for clinical researchers: Risk difference, risk ratio, and odds ratio. *Restor Dent Endod* **42**, 72–76 (2017).

23. Riecher-Rössler, A. Sex and gender differences in mental disorders. *The Lancet Psychiatry* **4**, 8–9 (2017).

24. McLean, C. P., Asnaani, A., Litz, B. T. & Hofmann, S. G. Gender Differences in Anxiety Disorders: Prevalence, Course of Illness, Comorbidity and Burden of Illness. *J Psychiatr Res* **45**, 1027–1035 (2011).

25. McNab, S. *et al.* Comment: silent burden no more: a global call to action to prioritize perinatal mental health. *BMC Pregnancy and Childbirth* **22**, 308 (2022).

26. Grey, I. *et al.* The role of perceived social support on depression and sleep during the COVID-19 pandemic. *Psychiatry Res* **293**, 113452 (2020).

27. Guo, K. *et al.* Assessing social support impact on depression, anxiety, and stress among undergraduate students in Shaanxi province during the COVID-19 pandemic of China. *PLoS One* **16**, e0253891 (2021).

For peer review only

28. Ortiz-Calvo, E. *et al.* The role of social support and resilience in the mental health impact of the COVID-19 pandemic among healthcare workers in Spain. *J Psychiatr Res* **148**, 181–187 (2022).

29. Długosz, P., Liszka, D. & Yuzva, L. The Link between Subjective Religiosity, Social Support, and Mental Health among Young Students in Eastern Europe during the COVID-19 Pandemic: A Cross-Sectional Study of Poland and Ukraine. *Int J Environ Res Public Health* **19**, 6446 (2022).

30. Miani, C., Wandschneider, L., Niemann, J., Batram-Zantvoort, S. & Razum, O. Measurement of gender as a social determinant of health in epidemiology—A scoping review. *PLoS One* **16**, e0259223 (2021).

31. Bolte, G. *et al.* Integrating Sex/Gender into Environmental Health Research: Development of a Conceptual Framework. *Int J Environ Res Public Health* **18**, 12118 (2021).

32. Short, S. E., Yang, Y. C. & Jenkins, T. M. Sex, Gender, Genetics, and Health. *Am J Public Health* **103**, S93–S101 (2013).

For peer review only

Figure legends:

**Figure 1: Percentage of participants who reported having depression, Nervousness, Loneliness, and Sleep Trouble by country.**

As shown in figure 1, the percentage of self-reported depression was over 40% in Portugal (46.44%), Bulgaria (42.5%), and Hungary (42.33%). Portugal (53.37%), Bulgaria (50.83%) also had the highest percentages of self-reported nervousness. The percentage of loneliness was highest in Greece (52.91%) and that of sleep trouble was highest in Estonia (49.13%).

For peer review only

**Figure 2: comparison of the total number of conditions reported by male and female participants.**

Figure 2 shows that about a quarter of the men and women reported at least one of the four conditions. However, the percentage of reporting more than one condition was higher among women than men. For example, 10.72% of the women reported having all four conditions compared with 4.81% among men.

For peer review only

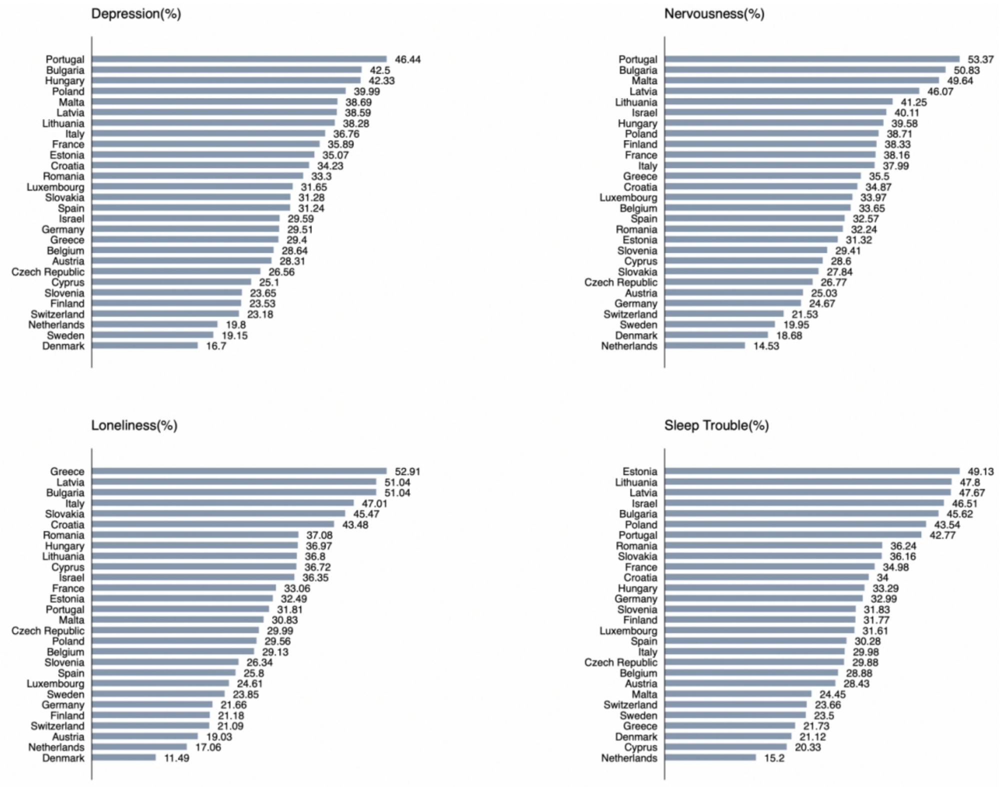


Percentage of participants who reported having depression, Nervousness, Loneliness, and Sleep Trouble by country.

165x129mm (220 x 220 DPI)

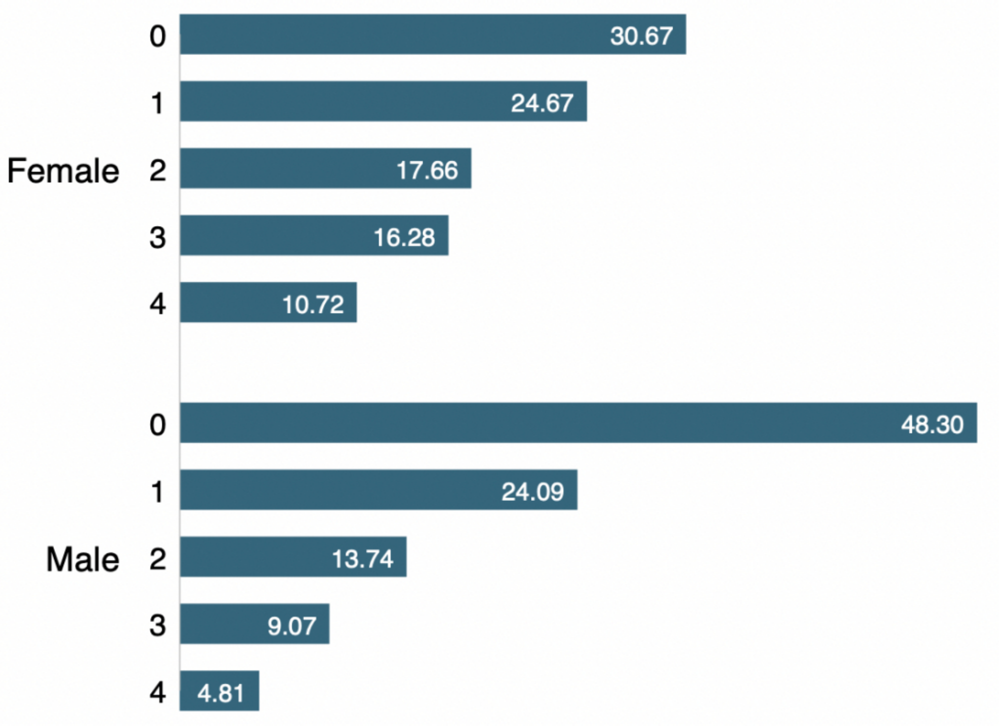


comparison of the total number of conditions reported by male and female participants.

118x86mm (220 x 220 DPI)

Appendix

Table 1a: Sample description (Share 8).

| age | Freq. | Percent | Cum. |
|---|---|---|---|
| 65-69 years | 9857 | 26.92 | 26.92 |
| 70-74 years | 9719 | 26.54 | 53.46 |
| 75-79 years | 7508 | 20.50 | 73.96 |
| 80-84 years | 5399 | 14.74 | 88.70 |
| 85+ | 4138 | 11.30 | 100.00 |
| | | | |
| Sex | Freq. | Percent | Cum. |
| Male | 15719 | 42.92 | 42.92 |
| Female | 20902 | 57.08 | 100.00 |
| | | | |
| Employment status | Freq. | Percent | Cum. |
| Retired | 32195 | 88.03 | 88.03 |
| Employment | 1626 | 4.45 | 92.48 |
| Unemployed | 79 | 0.22 | 92.69 |
| Permanently sick or disabled | 298 | 0.81 | 93.51 |
| Homemaker | 1980 | 5.41 | 98.92 |
| Other | 395 | 1.08 | 100.00 |
| | | | |
| Rating of subjective health | Freq. | Percent | Cum. |
| Excellent | 1266 | 3.46 | 3.46 |
| Very good | 4789 | 13.09 | 16.55 |
| Good | 14290 | 39.05 | 55.60 |
| Fair | 12106 | 33.08 | 88.69 |
| Poor | 4140 | 11.31 | 100.00 |
| | | | |
| Country | | | |
| Austria | 1943 | 5.31 | 5.31 |
| Germany | 1466 | 4.00 | 9.31 |
| Sweden | 856 | 2.34 | 11.65 |
| Netherlands | 592 | 1.62 | 13.26 |
| Spain | 1545 | 4.22 | 17.48 |
| Italy | 2528 | 6.90 | 24.38 |
| France | 1458 | 3.98 | 28.37 |

For peer review only

| | | | |
|---|---|---|---|
| Denmark | 1143 | 3.12 | 31.49 |
| Greece | 2515 | 6.87 | 38.36 |
| Switzerland | 1452 | 3.96 | 42.32 |
| Belgium | 2427 | 6.63 | 48.95 |
| Israel | 1132 | 3.09 | 52.04 |
| Czech Republic | 1808 | 4.94 | 56.98 |
| Poland | 1795 | 4.90 | 61.88 |
| Luxembourg | 581 | 1.59 | 63.46 |
| Hungary | 715 | 1.95 | 65.42 |
| Portugal | 860 | 2.35 | 67.76 |
| Slovenia | 2313 | 6.32 | 74.08 |
| Estonia | 3012 | 8.22 | 82.31 |
| Croatia | 1305 | 3.56 | 85.87 |
| Lithuania | 774 | 2.11 | 87.98 |
| Bulgaria | 480 | 1.31 | 89.29 |
| Cyprus | 490 | 1.34 | 90.63 |
| Finland | 855 | 2.33 | 92.97 |
| Latvia | 623 | 1.70 | 94.67 |
| Malta | 548 | 1.50 | 96.16 |
| Romania | 919 | 2.51 | 98.67 |
| Slovakia | 486 | 1.33 | 100.00 |

For peer review only

STROBE Statement—Checklist of items that should be included in reports of ***cross-sectional studies***

| | Item No | Recommendation |
|---|---|---|
| **Title and abstract** **(page 2)** | 1 | (*a*) Indicate the study's design with a commonly used term in the title or the abstract |
| | | (*b*) Provide in the abstract an informative and balanced summary of what was done and what was found |
| **Introduction** | | |
| Background/rationale **(page 3)** | 2 | Explain the scientific background and rationale for the investigation being reported |
| Objectives **(page 1, 4)** | 3 | State specific objectives, including any prespecified hypotheses |
| **Methods** | | |
| Study design **(page 4-5)** | 4 | Present key elements of study design early in the paper |
| Setting **(page 4)** | 5 | Describe the setting, locations, and relevant dates, including periods of recruitment, exposure, follow-up, and data collection |
| Participants **(page 4)** | 6 | (*a*) Give the eligibility criteria, and the sources and methods of selection of participants |
| Variables **(page 5)** | 7 | Clearly define all outcomes, exposures, predictors, potential confounders, and effect modifiers. Give diagnostic criteria, if applicable |
| Data sources/ measurement **(page 4)** | 8* | For each variable of interest, give sources of data and details of methods of assessment (measurement). Describe comparability of assessment methods if there is more than one group |
| Bias **(page 5)** | 9 | Describe any efforts to address potential sources of bias |
| Study size **(page 5)** | 10 | Explain how the study size was arrived at |
| Quantitative variables | 11 | Explain how quantitative variables were handled in the analyses. If applicable, describe which groupings were chosen and why |
| Statistical methods **(page 6)** | 12 | (*a*) Describe all statistical methods, including those used to control for confounding |
| | | (*b*) Describe any methods used to examine subgroups and interactions |
| | | (*c*) Explain how missing data were addressed |
| | | (*d*) If applicable, describe analytical methods taking account of sampling strategy |
| | | (*e*) Describe any sensitivity analyses |
| **Results** | | |
| Participants **(page 7)** | 13* | (a) Report numbers of individuals at each stage of study—eg numbers potentially eligible, examined for eligibility, confirmed eligible, included in the study, completing follow-up, and analysed |
| | | (b) Give reasons for non-participation at each stage |
| | | (c) Consider use of a flow diagram |
| Descriptive data **(page 7)** | 14* | (a) Give characteristics of study participants (eg demographic, clinical, social) and information on exposures and potential confounders |
| | | (b) Indicate number of participants with missing data for each variable of interest |
| Outcome data **(page 9-11)** | 15* | Report numbers of outcome events or summary measures |
| Main results **(page 9-11)** | 16 | (*a*) Give unadjusted estimates and, if applicable, confounder-adjusted estimates and their precision (eg, 95% confidence interval). Make clear which confounders were adjusted for and why they were included |


For peer review only - http://bmjopen.bmj.com/site/about/guidelines.xhtml

| | | |
|---|---|---|
| | | (*b*) Report category boundaries when continuous variables were categorized |
| | | (*c*) If relevant, consider translating estimates of relative risk into absolute risk for a meaningful time period |
| Other analyses | 17 | Report other analyses done—eg analyses of subgroups and interactions, and sensitivity analyses |
| **Discussion** | | |
| Key results **(page 11-12)** | 18 | Summarise key results with reference to study objectives |
| Limitations **(page 14)** | 19 | Discuss limitations of the study, taking into account sources of potential bias or imprecision. Discuss both direction and magnitude of any potential bias |
| Interpretation **(page 11-12)** | 20 | Give a cautious overall interpretation of results considering objectives, limitations, multiplicity of analyses, results from similar studies, and other relevant evidence |
| Generalisability **(page 14)** | 21 | Discuss the generalisability (external validity) of the study results |
| **Other information** | | |
| Funding **(page 15)** | 22 | Give the source of funding and the role of the funders for the present study and, if applicable, for the original study on which the present article is based |

*Give information separately for exposed and unexposed groups.

**Note:** An Explanation and Elaboration article discusses each checklist item and gives methodological background and published examples of transparent reporting. The STROBE checklist is best used in conjunction with this article (freely available on the Web sites of PLoS Medicine at http://www.plosmedicine.org/, Annals of Internal Medicine at http://www.annals.org/, and Epidemiology at http://www.epidem.com/). Information on the STROBE Initiative is available at www.strobe-statement.org.

For peer review only



For peer review only - http://bmjopen.bmj.com/site/about/guidelines.xhtml
